# Germline β−1,3-glucan deposits are required for female gametogenesis in *Arabidopsis thaliana*

**DOI:** 10.1038/s41467-024-50143-0

**Published:** 2024-07-12

**Authors:** Sara C. Pinto, Weng Herng Leong, Hweiting Tan, Lauren McKee, Amelie Prevost, Chao Ma, Neil. J. Shirley, Rosanna Petrella, Xiujuan Yang, Anna M. Koltunow, Vincent Bulone, Masahiro M. Kanaoka, Tetsuya Higashyiama, Sílvia Coimbra, Matthew R. Tucker

**Affiliations:** 1 LAQV REQUIMTE, Departamento de Biologia, Faculdade de Ciências, Universidade do Porto, rua do Campo Alegre s/n, 4169-007 Porto, Portugal; 2 Waite Research Institute, School of Agriculture, Food and Wine, University of Adelaide, Waite Campus, Urrbrae, SA 5064 Australia; 3 Australian Research Council Centre of Excellence in Plant Cell Walls, University of Adelaide, Urrbrae, SA 5064 Australia; 4 Department of Chemistry, Division of Glycoscience, KTH Royal Institute of Technology, Stockholm, Sweden; 5 Dipartimento di Bioscienze, Università Degli Studi di Milano, Via Celoria 26, 20133 Milan, Italy; 6 Centre for Crop Sciences, Queensland Alliance for Agriculture and Food Innovation, The University of Queensland, Brisbane, QLD 4072 Australia; 7 Institute of Transformative Bio-Molecules, Nagoya University, Furo-cho, Chikusa-ku, Nagoya, Aichi 464-8601 Japan; 8 Faculty of Bioresource Sciences, Prefectural University of Hiroshima, 5562 Nanatsuka-cho, Shobara City, Hiroshima 727-0023 Japan; 9 Department of Biological Sciences, Graduate School of Science, The University of Tokyo, 7-3-1 Hongo, Bunkyo-ku, Tokyo 113-0033 Japan; 10 Present Address: Department of Cell and Developmental Biology, John Innes Centre, Norwich Research Park, Norwich, NR4 7UH UK; 11 Present Address: College of Medicine and Public Health, Flinders University, Bedford Park Campus, Sturt Road, Bedford Park, SA 5042 Australia

**Keywords:** Seed development, Cell fate, Plant cell biology

## Abstract

Correct regulation of intercellular communication is a fundamental requirement for cell differentiation. In *Arabidopsis thaliana*, the female germline differentiates from a single somatic ovule cell that becomes encased in β−1,3-glucan, a water insoluble polysaccharide implicated in limiting pathogen invasion, regulating intercellular trafficking in roots, and promoting pollen development. Whether β−1,3-glucan facilitates germline isolation and development has remained contentious, since limited evidence is available to support a functional role. Here, transcriptional profiling of adjoining germline and somatic cells revealed differences in gene expression related to β−1,3-glucan metabolism and signalling through intercellular channels (plasmodesmata). Dominant expression of a β−1,3-glucanase in the female germline transiently perturbed β−1,3-glucan deposits, allowed intercellular movement of tracer molecules, and led to changes in germline gene expression and histone marks, eventually leading to termination of germline development. Our findings indicate that germline β−1,3-glucan fulfils a functional role in the ovule by insulating the primary germline cell, and thereby determines the success of downstream female gametogenesis.

## Abstract (web summary)

During female gametogenesis in plants, the Megaspore Mother Cell (MMC) accumulates β−1,3-glucan. Here Pinto et al. show that increased β−1,3-glucanase in the MMC delays β−1,3-glucan deposition, disrupts cell identity, and halts gametogenesis.

## Introduction

Female germline development in Arabidopsis, like most seed-bearing plants, gives rise to a haploid gametophyte in the ovule that is fertilised to initiate seed formation. This process is fundamental to plant reproduction and incorporates two phases: sporogenesis and gametogenesis. During sporogenesis, a diploid sporophytic (somatic) cell termed the megaspore mother cell (MMC) differentiates at the tip of the ovule and expands rapidly compared to the surrounding cells^1^. During expansion, the MMC is distinguished from surrounding cells via prominent β−1,3-glucan (callose) deposits in the cell wall^2^, as well as an enlarged central nucleus, unique histone marks^3^, and a specific gene expression profile^4^. The MMC is the only ovule cell to enter meiosis, producing four haploid callose-encased megaspores. A single megaspore (functional megaspore; FM) is selected to initiate the mitotic events of gametogenesis, which coincides with removal of callose from the cell wall^2^. Gametogenesis is characterised by three syncytial mitoses, followed by cellularisation and differentiation, and ends with the production of a mature female gametophyte containing an egg cell, a central cell, and five accessory cells. The early events of germline development are therefore defined by prominent yet transient callose deposition, whereby callose accumulates in the MMC and megaspores but is lost in the cell wall of the FM as it enters mitosis.

The Arabidopsis ovule primordium is divided into three domains along a proximal-distal axis^5^. The most distal of these domains is the nucellus, which gives rise to the female germline and incorporates a range of epidermal and sub-epidermal cell-types^6^. Interactions between these cell types are complex^1,7^. A recent morphometric study concluded that region-specific growth-promoting signals and physical constraints between cells are required to canalise MMC development^8^. Moreover, multiple genes required for sporogenesis are expressed outside of the germline, leading to models of germline development that incorporate non-cell autonomous signalling^1^. Predicted molecular components of this model include members of the *RNA-DIRECTED DNA METHYLATION* (*RdDM*) pathway^9^ and transcriptional regulators such as *SEEDSTICK* (*STK*)^10^, *WUSCHEL* (*WUS*)^11^ and *SPOROCYTELESS/NOZZLE* (*SPL*)^10,12^.

One gap in this non-cell autonomous model is reconciling the requirement for external developmental cues with the presence of a callose-rich cell wall surrounding the MMC and megaspores. Callose is a water-insoluble polymer^13^. During pathogenesis and root development, callose can inhibit signalling by physically reinforcing infection sites, blocking access to receptors that mediate apoplastic signalling, or filling the neck regions of intercellular channels called plasmodesmata (PD) to physically regulate the symplastic flow of RNAs, sugars and proteins between adjoining cells^14^. The abundance of callose in the MMC wall favours a model whereby the cell is isolated from external signals, and can initiate specialised germline programs. Consistent with this isolation model, PD are initially evident in the MMC wall^15^ but appear to become non-permissive over time. A mobile GFP tracer expressed from a phloem-specific *pSUC2:GFP* transgene was initially detected in all ovule cells but was gradually depleted from the expanding MMC^16^, coinciding with callose accumulation. Despite this, it remains unclear whether callose fulfils any functional role during female germline development, or is a redundant artefact of ovule evolution. This quandary was first raised in classical studies of ovule development^17^ and has remained a long-standing question in female germline development.

Here we assess the transcriptional landscape of the MMC and surrounding cells, and test the functional requirement for callose deposition during female germline development. Analysis of high-resolution transcriptomes of the MMC and nucellus revealed differential expression of genes involved in callose metabolism, intercellular communication and PD function. Cell-type specific expression of a functional β−1,3-glucanase perturbed germline development, de-regulated movement of mobile fluorescent tracers, and induced specific changes in the expression of MMC-enriched genes involved in chromatin architecture, meiosis and cell cycle transition. This coincided with transient defects in callose accumulation and changes in histone marks in the MMC, suggesting an inability to correctly maintain germline fate. Our data indicate that callose fulfils a critical role in MMC development and determines the success of downstream female gametogenesis.

## Results

### Transcriptional analysis of germline and somatic cells reveals distinct gene expression profiles

**Fig. 1:**
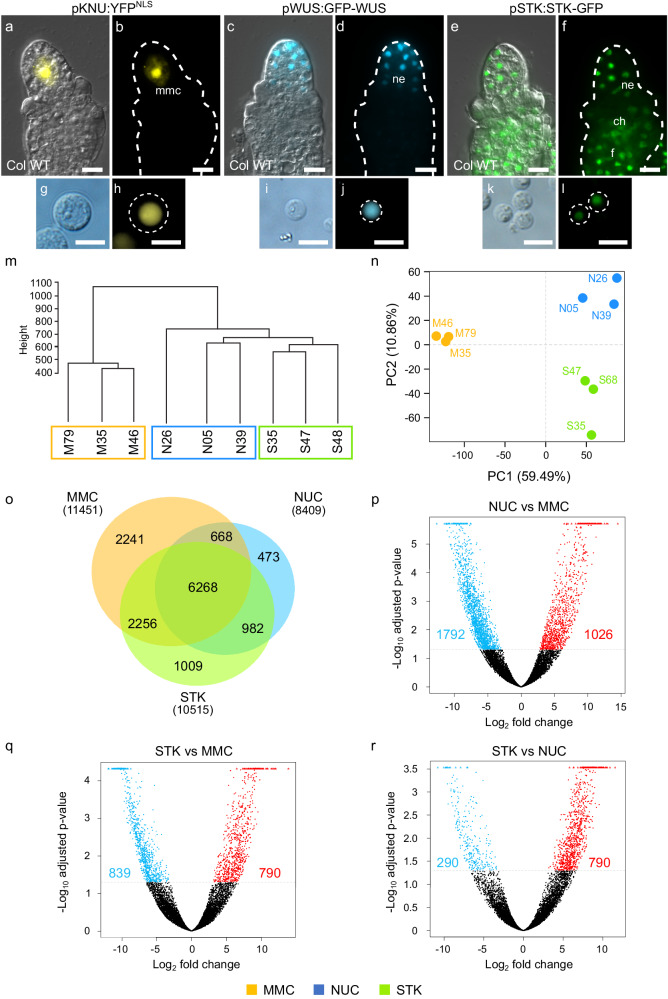
Cell-type specific transcriptional profiling of young Arabidopsis ovules. **a**, **b** *pKNU:YFP*^*NLS*^ ovule, the yellow fluorescent protein is detected solely in the megaspore mother cell (mmc) nucleus. **c**, **d** *pWUS:GFP-WUS* ovule, the WUS-GFP fusion protein is detected in the nucellus epidermis (ne). **e**, **f** *pSTK:STK-GFP* reporter protein is observed in the somatic cells of the ovule, in the nucellus epidermis (ne), chalaza (ch) and funiculus (f). Protoplasts produced from *pKNU:YFP*^*NLS*^ (**g**, **h**), *pWUS:WUS-GFP* (**i**, **j**), and *pSTK:STK-GFP* (**k**, **l**) ovules showing fluorescent protein expression restricted to the nucleus. **a**, **c**, **e** Images result from merging bright-field DIC with YFP/GFP fluorescence. **b**, **d**, **f** Fluorescence channel only. Scale bars: 10 μm. RNAseq quality assessment: **m** Hierarchical clustering dendrogram and **n** Principal Component Analysis (PCA) scatter plot using read count matrixes calculated by DESeq2 method. **o** Venn diagram showing the number of genes expressed in each cell type (in parenthesis) and the overlap between transcriptomes. **p**–**r** Volcano plots depicting the differentially expressed genes for each comparison (blue: downregulated genes = FDR < 0.05, log_2_ (fold change) < −2; red: upregulated genes = FDR < 0.05, log_2_ (fold change) > 2). y axis - adjusted *p*-value is the FDR value calculated as described in the methods section. M35, M46, M79 = *pKNU:YFP*^*NLS*^ samples (MMC) biological replicates; N05, N26, N39 = *pWUS:GFP-WUS* (NUC) biological replicates; S35, S47, S68 = *pSTK:STK-GFP* (STK) biological replicates.

To explore the molecular pathways that contribute to female germline development we generated and characterised different ovule cell type-specific transcriptomes. We used marker lines that define the MMC (*pKNU:YFP*^*NLS*^^18^; Fig. 1a, b), nucellus (*pWUS:GFP-WUS*^18^; Fig. 1c, d) and most somatic cells of the ovule (*pSTK:STK-GFP*^19^; Fig. 1e, f) coupled with sorting of fluorescent protoplasts. Protoplasts were isolated from ovules at stage 2-II when callose starts to accumulate around the expanding MMC. Enzymatic separation of the ovule cells produced *pKNU:YFP*^*NLS*^ protoplasts that varied in diameter from 8 to 14 µm (hereafter termed the “MMC” sample; Fig. 1g, h). By comparison, *pWUS:GFP-WUS* (“NUC” sample) and *pSTK:STK-GFP* protoplasts (“STK” sample) varied in size from 5 to 8 µm in diameter (Fig. 1i–l). Only protoplasts showing strong nuclear signals were collected for RNA extraction (Fig. 1h, j, l). Using these stringent parameters, yields were low but consistent. For example, we typically obtained around 4 intact, fluorescent *pKNU:YFP*^*NLS*^ protoplasts per 40 ovules (i.e. the average number of ovules per flower).

RNA extraction from 20 to 40 protoplasts per biological replicate and subsequent RNA sequencing generated on average 7.5 million reads per sample, and over 70% of these reads were uniquely mapped to the Arabidopsis TAIR10 genome (Supplementary Table 1). To investigate whether the transcriptomic profiles were distinguishable by cell type, unsupervised hierarchical clustering and principal component analysis (PCA) were conducted. The clustering analysis showed that the MMC biological replicates form a cluster independent of NUC and STK replicates (Fig. 1m). Regarding PCA, two components explain most of the variability and underlie the separation of the replicates into three groups according to their cell type (Fig. 1n). While PC1 separates the MMC samples from the samples of somatic origin, PC2 allows separation of the NUC and STK samples. In summary, both analyses show that the transcriptomic profiles are distinguishable based on sample origin and, importantly, a clear distinction is detected between the germline and somatic transcriptomes.

The number of expressed genes was similar across transcriptomes, ranging from 8400 to 11500 genes (Fig. 1o). Of these, 6268 genes were commonly expressed in the three samples. Differential gene expression analysis (Supplementary Dataset 1) revealed the highest number of differentially expressed genes (DEGs) between NUC and MMC, i.e., 2818 genes (Fig. 1p). The comparison between STK and MMC showed a total of 1629 DEGs (Fig. 1q), whereas the lowest number of DEGs were identified between STK and NUC, with a total of 1288 genes (Fig. 1r). Subsequent analysis of the DEGs revealed cell-type specific expression profiles consistent with previous published transcriptomic data, in situ hybridisation experiments, reporter lines and/or immunolocalisation experiments (Supplementary Fig. 1)^3,4,18–32^. Highly restricted expression patterns were confirmed for genes expressed solely in the MMC (Supplementary Fig. 1a, f, g), the nucellus (Supplementary Fig. 1b), and within the somatic domain defined by STK protoplasts (Supplementary Fig. 1c, d). We could also demonstrate that transcripts of genes known to be expressed throughout the ovule were detected in all three transcriptomes (Supplementary Fig. 1e). A direct comparison of our data, generated from fluorescence-sorted protoplasts, to the recently published unsupervised scRNAseq^6^ of young Arabidopsis ovule protoplasts (Supplementary Table 2) showed overlaps in the MMC cluster. This provided confidence that our method can be used to extract key differences between specific ovule cell types, such as the MMC and adjoining nucellus.

### Gene set enrichment analyses highlight differences in plasmodesmal signalling between ovule cell types

**Fig. 2:**
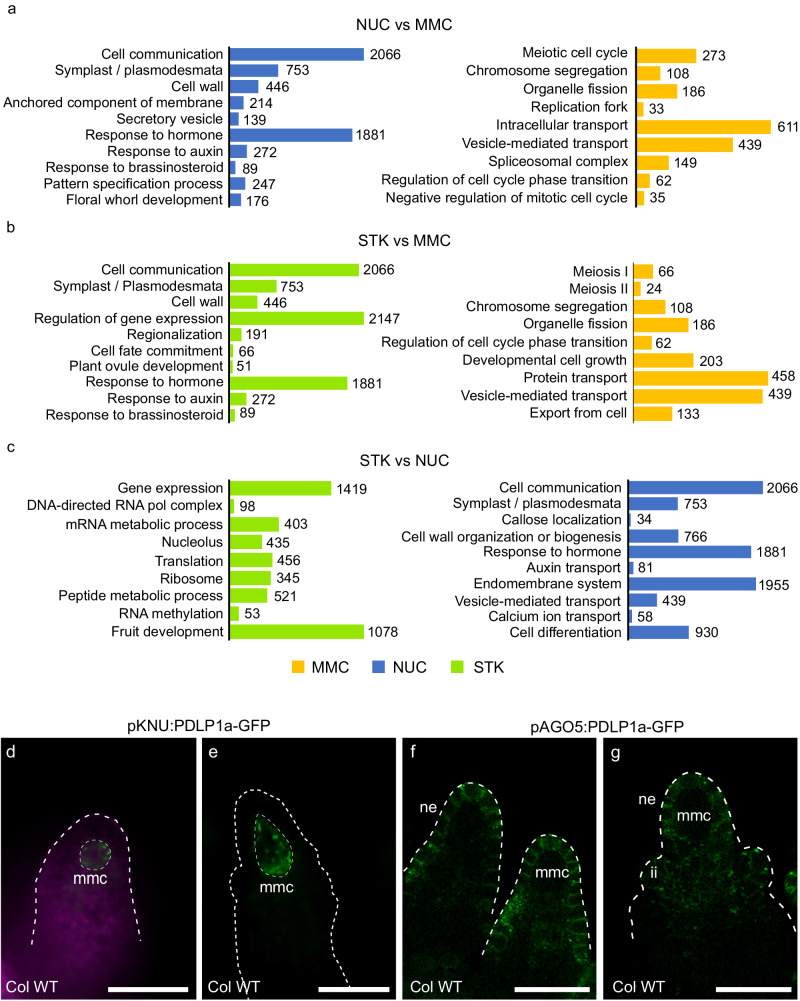
Enriched gene ontology terms in MMC, nucellar (NUC) and somatic (STK) transcriptomes. Selection of the biological processes and cell component GO terms comparing NUC and MMC (**a**), STK and MMC (**b**), and STK and NUC samples (**c**). Numbers at the end of the bars indicate the number of genes for each GO term. **d**, **e** pKNU:PDLP1a-GFP is expressed in a punctate pattern in the megaspore mother cell (mmc) walls. In **d**, purple colour shows autofluorescence. **f**, **g** pAGO5:PDLP1a-GFP is observed in the nucellar epidermis (ne) and inner integument (ii) cells. **d**, **e** Widefield fluorescence microscopy. **f**, **g** Laser Scanning Confocal Microscopy, max-projection of three GFP slices (green). Experiments were repeated at least 4 times for **d**, **e**, and three times for **f**, **g**, with similar results and representative micrographs are shown. Scale bars = 20 μm.

To define processes occurring in each cell type, a Gene Set Enrichment Analysis was performed for each sample pair combination (see Fig. 2 for a selection of relevant GO terms and Supplementary Dataset 2 for full lists of GO terms).

In comparison to the other samples, the MMC transcriptome clearly showed enrichment of terms related to meiosis, negative regulation of mitosis, cell cycle checkpoint, and vesicle-mediated transport. Relative to NUC, the MMC also showed enriched terms such as gene expression (transcription, translation and maturation) and RNA splicing (Fig. 2a; Supplementary Dataset 2). Conversely, relative to STK there were enriched terms in the MMC related to protein transport, secretion, and cell growth (Fig. 2b).

In the NUC transcriptome we found enriched GO terms related to cell communication, cell wall, signal transduction, plasmodesmata and symplast, compared to the MMC and STK samples (Fig. 2a, c). Additional terms related to cell differentiation and the endomembrane system, such as Golgi apparatus and endoplasmic reticulum, appeared when comparing NUC with STK, but not with the MMC (Fig. 2c; Supplementary Data 2). The NUC and MMC comparison uncovered terms associated with cell wall organisation and biogenesis (Fig. 2a; Supplementary Data 2), while the STK samples were always enriched for terms related to gene expression (Fig. 2b, c; Supplementary Data 2). Additionally, in the STK and MMC comparison, we detected terms related to cell wall, cell communication, response to hormone (namely auxin and brassinosteroid), plant ovule development, plasmodesma, and symplast (Fig. 2b). This shows some overlap with the NUC transcriptome, which is not unexpected considering the STK transcriptome also contains nucellar cells.

These results confirm that the overall molecular signatures are distinct and characteristic of each cell type. They also reveal that symplastic intercellular signalling pathway genes are enriched in the “nucellus” transcriptome relative to the adjoining germline cells. Indeed, from the 2818 genes differentially expressed between MMC and NUC, 267 gene transcripts are predicted to encode proteins that localise to PD^33^, accounting for about 9% of all DEGs. Because little is known about the regulation of symplastic transport components in ovules, we explored this pathway in greater detail.

### Genes related to PD composition and function are expressed in specific ovule cell types

First, we considered the location of PD in the ovule tissues of interest. PLASMODESMATA-LOCALIZED PROTEIN1a^34^ has been extensively used as a PD marker when fused to GFP (PDLP1a-GFP^35,36^). Importantly, PDLP1a is not normally expressed in the ovule (Supplementary Fig. 2), hence it forms a useful marker protein that is unlikely to interfere with endogenous PDLP1a activity. Expression of PDLP1a-GFP under the *KNU* promoter (*pKNU:PDLP1a-GFP*), which is exclusively detected in the MMC, showed a weak but punctate localisation pattern at the cell periphery, and was most abundant at the proximal pole (Fig. 2d, e). A *pAGO5:PDLP1a-GFP* construct directed GFP expression to the nucellar epidermis and inner integument primordia, and consistent with previous marker studies, was absent from the MMC (Fig. 2f, g). In the epidermis, PDLP1a-GFP protein was predominantly detected in anticlinal walls and assumed a punctate pattern consistent with PD localisation. PDLP1a-GFP was not obvious in the innermost wall of the epidermal cells that adjoin hypodermal cells, including the MMC at the distal tip. These data support previous TEM studies that suggest the MMC and NUC both contain PD^15^.

Next, we investigated candidate genes influencing PD composition and connectivity. Glucan synthases (GSLs) and β−1,3-glucanases (BGs) control the synthesis and hydrolysis of callose at PD^37^ and are key determinants of the PD size exclusion limit (SEL) that influences intercellular movement. Additionally, the receptors encoded by *PDLP*s and *PLASMODESMATA CALLOSE BINDING PROTEINS* (*PDCB*s) localise in membranes and are thought to promote callose deposition^14^. Most of these putative regulators of PD permeability showed restricted expression in the germline or somatic transcriptomes (Supplementary Figs. 2 and 3). For example, expression of *GSL2* was confined to the MMC (Supplementary Fig. 3a–c), while *GSL4* was detected in ovule cells other than the MMC (Supplementary Fig. 3a, d, e). *PDLP* and *PDCB* transcripts were detected only in the NUC and STK transcriptomes, consistent with the enrichment of symplastic pathways in those cell types (Supplementary Fig. 2).

Notably, the proportion of BGs and GSLs expressed in the MMC varied when compared to surrounding cells (Supplementary Fig. 3). Six out of nine expressed GSLs were abundant in the MMC. Of these, *GSL1*, *GSL5* and *GSL10* are predicted, and *GSL8* is confirmed, to localise to PD^33^. *GSL3* and *GSL6* showed elevated expression in NUC and STK and are also predicted to locate to PD^33^. By contrast, only three out of 13 detected BG were transcriptionally enriched in the MMC. Most of the BG genes were expressed specifically in the NUC/STK sample (Supplementary Fig. 3), with *AT1G66250*, *AT3G13560*, *AT2G01630* previously confirmed, and *AT4G29360*, *AT3G55430*, *AT3G07320* predicted, to be located in PD^33,38^.

Taken together, the cell-specific transcriptional profiles suggest that genes involved in callose biosynthesis, callose hydrolysis, and PD permeability are abundant and differentially expressed in the MMC compared to surrounding cells.

### Intercellular movement assays confirm the existence of a germline-specific symplastic domain in the ovule

**Fig. 3:**
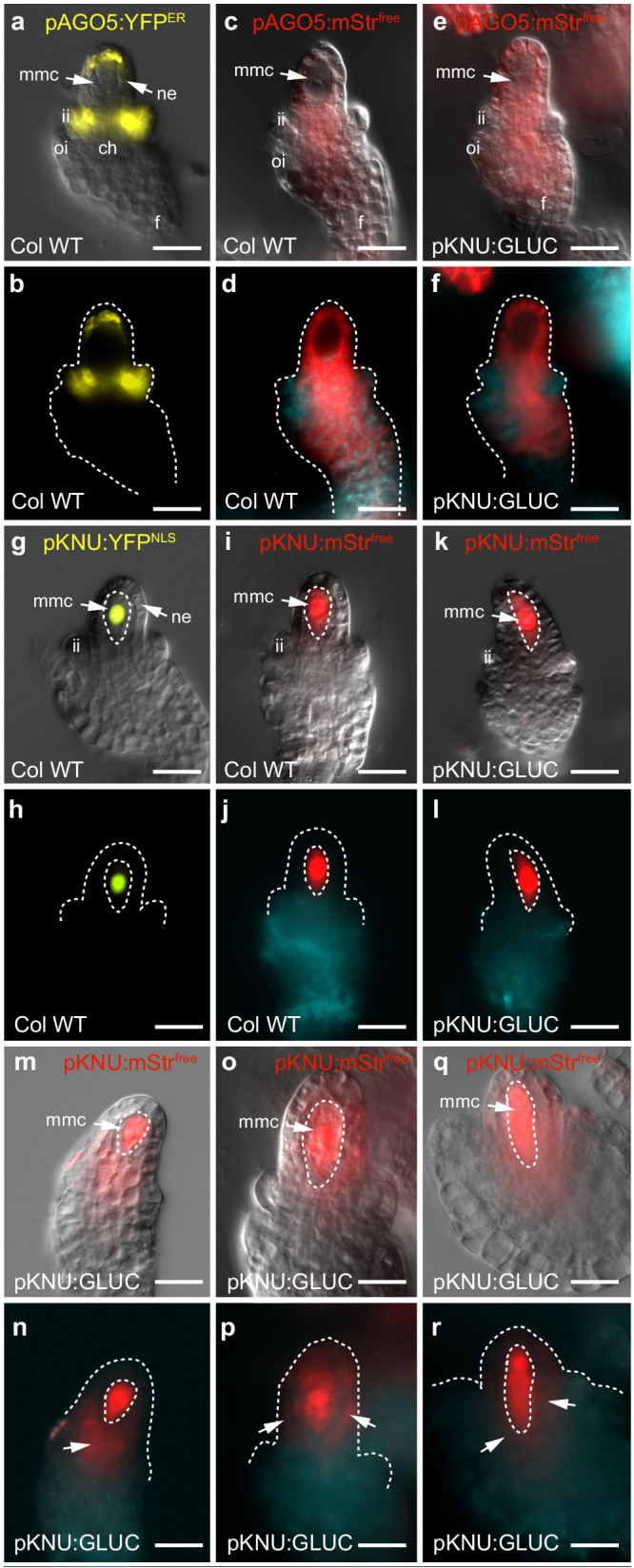
Localisation of cell autonomous and mobile fluorophores in wild-type and *pKNU:GLUC* ovules. **a**, **b** In wild-type ovules, cell-autonomous pAGO5:YFP^ER^ accumulates in the nucellar epidermis and inner integuments. **c**, **d** pAGO5:mStr^free^ shows a broader pattern in the chalaza and nucellus but is excluded from the MMC and is absent in the inner integuments. **e**, **f** A similar pattern is observed in *pKNU:GLUC* ovules. **g**, **h** In wild-type ovules, cell-autonomous pKNU:YFP^NLS^ accumulates in the nucleus of the MMC. **i**, **j**. pKNU:mStr^free^ spreads throughout the MMC but does not spread into the nucellar epidermis of wild-type ovules. **k**, **l** A similar pattern is observed in *pKNU:GLUC* ovules in the majority of cases. **m-r** In approximately 18% of *pKNU:GLUC* ovules, pKNU:mStr^free^ signal is apparent outside of the MMC in adjoining nucellus and chalaza cells (arrows in **n**, **p** and **r**). Experiments were repeated on 5 independent occasions with similar results. Representative micrographs are shown. Scale bars = 20 µm. ch chalaza, f funiculus, ii inner integument, mmc megaspore mother cell, ne nucellar epidermis, oi outer integument.

The expression of multiple GSL genes in the MMC coincides with a stage when the MMC becomes symplastically isolated from long-distance pSUC2:GFP moving into the ovule from the phloem^16^. However, whether the inhibition of movement depends on callose accumulation, or has any implications for germline development, remains unclear. To address this further, we aimed to develop a system for tracking molecule movement in and out of the MMC. Initially we tested the previously described long-distance pSUC2:GFP system for its ability to unload GFP from the phloem into the ovule. Unfortunately, we were unable to replicate pSUC2:GFP movement in our conditions. Instead, we developed cell-type specific markers to examine the details of local symplastic connectivity between the germline and surrounding cells. A gene encoding a mobile mStrawberry protein (mStr^free^) was expressed under the control of the *pAGO5* and *pKNU* promoters. In wild-type (WT) plants, the cell-autonomous endoplasmic reticulum (ER)-localised pAGO5:YFP^ER^ protein is unable to move between cells and accumulates in the nucellar epidermis, inner integument and chalaza, but is excluded from the MMC and funiculus (Fig. 3a, b^18^). In plants expressing *mStr*^*free*^ under the same *pAGO5* promoter (*pAGO5:mStr*^*free*^), a similar but broader fluorescent signal was observed. Apart from being detected in the nucellar epidermis and chalaza, the mStr^free^ signal was observed near the proximal funiculus but not in the inner integument or the MMC (Fig. 3c, d). This confirms that the mStr^free^ protein can move in a distal to proximal direction in the ovule, but cannot enter the MMC at the stage when callose deposits are present. Conversely, expression of nuclear localised (NLS) cell-autonomous YFP^NLS^ protein from the *pKNU* promoter was detected in the MMC, but not the nucellus (Fig. 3g, h^18^). In WT plants, mobile pKNU:mStr^free^ accumulated to high levels in the MMC (Fig. 3i, j), and, similar to the cell-autonomous pKNU:YFP^NLS^ protein, did not spread into surrounding nucellar cells, confirming symplastic isolation of the MMC.

**Fig. 4:**
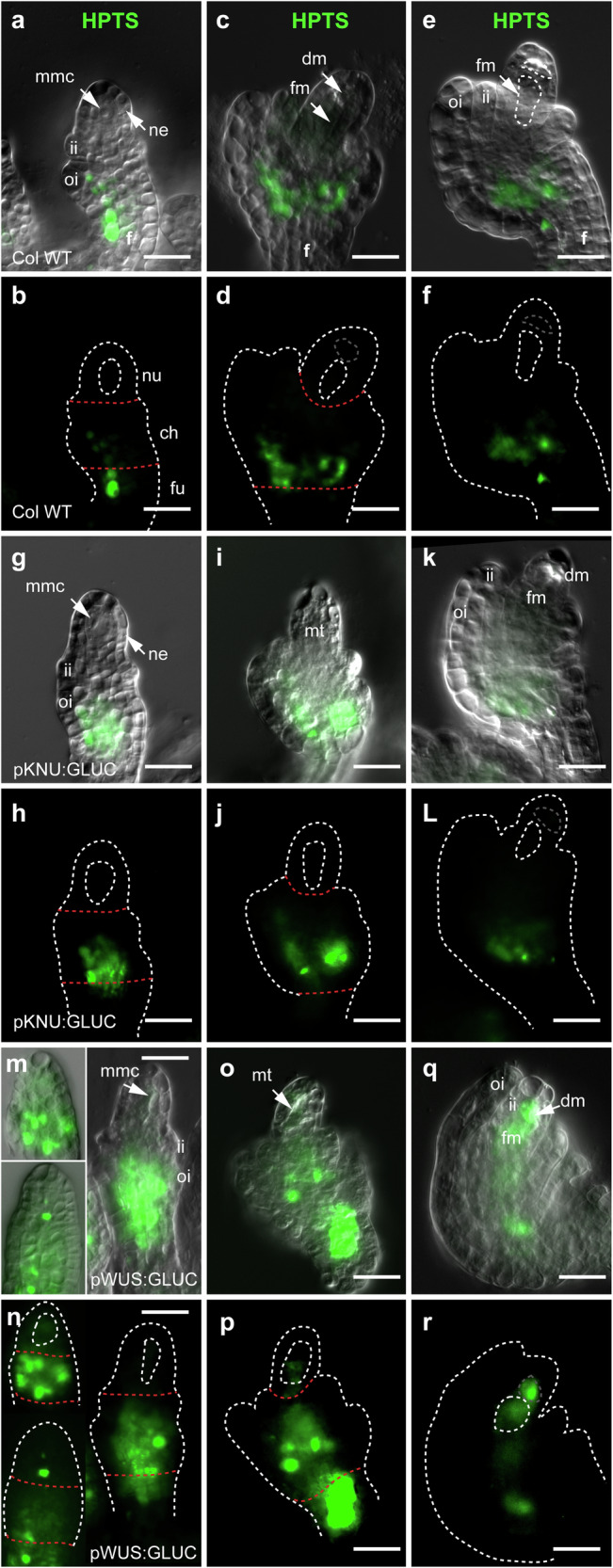
Localisation of HPTS tracer in wild-type, pKNU:GLUC and pWUS:GLUC ovules. **a**–**f** In wild-type, HPTS accumulates in the funiculus and chalaza of pre-meiotic and post-meiotic ovules. **g**–**l** A similar localisation pattern is observed in pKNU:GLUC ovules. **m**–**r** In pWUS:GLUC ovules, HTPS accumulates strongly in the funiculus and chalaza, progressing further towards the nucellus. Unlike the other tested genotypes, signal is occasionally detected in the vicinity of the mmc/megaspores. The red dashed lines indicate the interface between different ovule domains. Experiments were repeated on 4 independent occasions with similar results. Representative micrographs are shown. Scale bars = 20 µm. ch chalaza, dm degenerating megaspores, fu funiculus, ii inner integument, mmc megaspore mother cell, mt megaspore tetrad, ne nucellar epidermis, nu nucellus. oi outer integument.

Next, we considered the possibility that movement across the MMC-NUC interface is influenced by a stringent PD size exclusion limit. Previous studies have used 8-hydroxypyrene-1,3,6-trisulfonic acid trisodium salt (HPTS) as a mobile tracer dye to assess symplastic connectivity^16,39,40^, and in contrast to the ~27 kDa mStr^free^ protein, HPTS is only around 0.5 kDa in size. Intercellular movement of HPTS into ovules was tested using dissected inflorescence stems from WT plants. HPTS moved rapidly into the stem and upwards into flowers. In WT ovules, HPTS moved into the ovule and accumulated in defined spots within the funiculus and chalaza during germline development but was not detected in the nucellus or germline cells (Fig. 4a–f).

Overall, these mobility assays confirm a degree of symplastic connectivity between ovule cells, and symplastic barriers at the chalaza-funiculus and the germline-nucellus boundaries. The findings are consistent with earlier studies and provide new tools to investigate genes and pathways influencing symplastic connectivity in the ovule.

### Identification of an atypical β−1,3-glucanase that alters callose deposition in vitro and in vivo

**Fig. 5:**
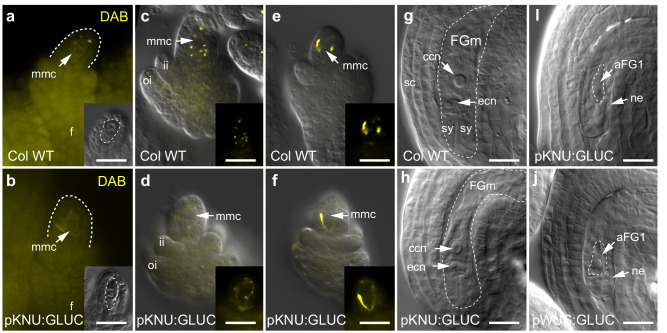
Callose deposition and phenotypes in ovules expressing GLUC. **a**, **c**, **e** In wild-type, decolourised aniline blue (DAB) stains punctate callose deposits in the wall of the MMC as it expands from stages 2-II to 2-III. These deposits accumulate over time and become large aggregates that encompass much of the MMC wall. **b**, **d**, **f** In *pKNU:GLUC* ovules, punctate callose deposits are less obvious in the MMC wall and the overall pattern is more diffuse compared to wild-type. Despite this, large aggregates are detected at the cell periphery at stage 2-III prior to the initiation of meiosis. **g** Cleared wild-type ovule containing a wild-type mature embryo sac at anthesis. **h** Example of a wild-type-like embryo sac from a *pKNU:GLUC* line. **i** Aborted FG1 embryo sac (where the functional megaspore has aborted gametogenesis at FG1 with a central nucleus and vacuoles at both poles) in a *pKNU:GLUC* ovule. **j** Aborted FG1 embryo sac in a pWUS:GLUC ovule. Staging according to Schneitz et al.^5^: (**a**, **b**) stage 2-I, (**c**–**f**) stage 2-II to 2-III. Experiments were repeated on 6 independent occasions with similar results. Representative micrographs are shown. Scale bars = 20 µm. aFG1 aborted female gametophyte, ccn central cell nucleus, ecn egg cell nucleus, DAB decolourised aniline blue, FGm mature female gametophyte, fu funiculus, ii inner integument, mmc megaspore mother cell, ne nucellar epidermis, oi outer integument, sc seed coat, sy synergid.

Callose is a classic marker for the MMC and megaspore tetrad and can be detected using decolourised aniline blue (DAB) and/or immunolabelling^41^. Consistent with the cell-specific gene expression data, WT ovules containing expanding MMCs (stage 2-II) showed callose accumulation in the MMC wall as spots and occasionally in the cell plate of adjoining nucellar cells (Fig. 5a). When the MMC was fully expanded (stage 2-III), DAB staining revealed larger aggregates at the cell periphery that eventually encompassed the entire cell wall (Fig. 5c, e). During meiosis, callose was most abundant in walls separating the megaspores, and became concentrated in the degenerating megaspores, at the base of the FM, prior to the initiation of gametogenesis (Supplementary Fig. 4a). No DAB staining was detected in the developing gametophyte where mitosis occurs in the absence of cytokinesis (Supplementary Fig. 4b, c).

To address whether callose deposition in the MMC is required to enforce its symplastic isolation, and whether this is needed for germline development, we used several approaches. First, we examined plants carrying mutations in the MMC-enriched *GSL* genes, but found that in our growing conditions, callose was still present in the MMC wall. We speculate that complete inhibition of callose biosynthesis in the MMC may require a higher-order cell-type-specific *gsl* mutant. Another strategy to modify callose levels involves ectopic-expression or mutation of BG gene, which has previously been reported to decrease or increase callose deposition, respectively, affecting cell-to-cell movement of molecules, as well as growth, development, and fertility^42–45^. We therefore considered whether cell-type specific expression of a BG might be sufficient to disturb callose accumulation and the germline-nucellus symplastic barrier. To avoid potential problems with endogenous gene silencing in Arabidopsis, we searched for suitable BG genes from other species. Previous reports described the *GLUC* gene (Supplementary Fig. 5a, b) as a putative BG from *Hieracium piloselloides* that is expressed during megasporogenesis^46^ and shares homology with the Arabidopsis anther-specific At4g14080 (A6) and At3g23770 (A6-like1; A6-L1) genes (Supplementary Fig. 5a). The A6/GLUC sequences reside within the α-clade of the GH17 BG family^47^ and contain an N’-terminal signal peptide but lack a glycosylphosphatidylinositol (GPI)-anchor. In contrast to the Arabidopsis A6-like proteins, the predicted GLUC amino acid sequences from *Hieracium* and other Asteraceae species also lack a C’-terminal X8 carbohydrate-binding domain (Supplementary Fig. 4b, 5a). Thus, *GLUC* is similar but not identical to BGs associated with reproductive callose dissolution in Arabidopsis.

To test the enzymatic activity of GLUC, the protein was expressed in *E. coli*. A filtrate of control and GLUC expressing cultures was incubated with a range of substrates including cellohexaose, laminarihexaose, xyloglucan, 1,3;1,4-β-glucan, lichenan, curdlan, laminarin and yeast β-glucan. Incubation with GLUC led to the release of glucose from 1,3-β-glucan substrates that contain 1,3-linkages only, but was unable to cleave 1,3-linkages in substrates such as barley 1,3;1,4-β-glucan (Supplementary Fig. 5c). This suggests that *GLUC* encodes a functional BG.

Next, we investigated the sub-cellular location of GLUC, which was predicted to be extracellular based on DeepLoc 2.0^48^. The coding sequence was fused to GFP and expressed in *Allium cepa* (onion) and *Nicotiana benthamiana* (tobacco) epidermal cells using the constitutive CaMV 35 S promoter. In onion cells, GLUC-GFP accumulated in strands of ER located around the nucleus and throughout the cell (Supplementary Fig. 6e). GLUC-GFP was also located in punctate spots at the cell periphery adjoining the plasma membrane, indicating that the enzyme is likely to be secreted and may accumulate in PD (Supplementary Fig. 6f, g). This location closely resembled that of other proteins previously confirmed to accumulate and function in PD^49^. GLUC-GFP localisation was also examined in tobacco leaf pavement cells, where PD are easily identified by DAB staining of callose^50^. Approximately 50% of the punctate spots labelled with DAB also showed GLUC-GFP signal (Supplementary Fig. 6h–j), suggesting that GLUC co-locates at least partially with the PD. Quantification of punctate callose deposits indicated that cells expressing 35S:GLUC-GFP or 35S:GFP-GLUC showed a reduced frequency of DAB staining relative to plants expressing 35S:GFP (Supplementary Fig. 6k). In agreement with the in vitro assays of the recombinant GLUC enzyme, these results demonstrate that GLUC is able to hydrolyse PD callose in vivo.

To address the effects of GLUC expression on callose deposition in the ovule, two constructs were generated; a germline-specific *pKNU:GLUC* gene to target the germline-nucellus symplastic block, and a nucellus-specific *pWUS:GLUC* gene, with a broader zone of action including the whole nucellar epidermis adjoining the chalaza. Ovules from transgenic plants were analysed by DAB staining in comparison to WT.

DAB staining patterns in *pWUS:GLUC* ovules appeared similar to WT with regards to the timing and amount of callose in the MMC wall, although the labelling occasionally appeared diffuse at stage 2-II (compare Supplementary Figs. 7a and 5c). Callose was also detected in ovules from *pKNU:GLUC* plants, but fluorescence intensity measurements confirmed a significant reduction in callose accumulation in the MMC compared to WT (Supplementary Fig. 8). Detailed analysis revealed that the appearance of punctate callose deposits in the MMC wall at ovule stage 2-II was consistently delayed (Fig. 5b). This was also confirmed by immunolabelling with an anti-callose antibody (BS400-2; Supplementary Fig 9). Thin sections of ovules from WT plants highlighted punctate callose deposits in the wall between the MMC and adjoining nucellar epidermal cells at stages 2-I and 2-II (Supplementary Fig. 9a–c, e). By contrast, in ~40% (*n* = 63 ovules) of the *pKNU:GLUC* ovule sections that contained an MMC, callose deposits were not detected or infrequently detected in the MMC wall (Supplementary Fig. 9d, f). Although the callose labelling was initially reduced or delayed in *pKNU:GLUC* ovules (Fig. 5d), accumulation of other cell wall components, as detected by the LM20 (methylesterified pectin)^51^ and JIM13 (arabinogalactan proteins)^52^ antibodies, appeared unchanged. Moreover, callose was eventually detected in the MMC wall of all ovules. Indeed, apart from the early differences, the subsequent stages of megasporogenesis in both *pKNU:GLUC* and *pWUS:GLUC* showed similar patterns of callose deposition and DAB staining intensity to WT (Supplementary Fig. 4d–f; Supplementary Fig. 7c, d; Supplementary Fig. 8). Taken together, these results suggest that callose deposition in the MMC wall is transiently modified in *pKNU:GLUC* ovules during MMC expansion. Defects in callose deposition were less obvious in *pWUS:GLUC* lines, and were not investigated further here.

### Cell type-specific expression of GLUC leads to defects in female gametogenesis and local changes in symplastic connectivity

The *pKNU:GLUC* and *pWUS:GLUC* lines were indistinguishable from WT in terms of plant height and growth habit. However, analysis of ovule development indicated that both constructs compromised female gametogenesis (Fig. 5g–j; Supplementary Table 3). In WT, approximately 98% (*n* = 3335) of the ovules at anthesis contained a mature female gametophyte, including an egg cell and central cell nucleus (Fig. 5g). At the same stage of development, ~39% of the ovules in *pKNU:GLUC* lines (*n* = 3288) had aborted at the first stage of gametogenesis (FG1; Fig. 5i; Supplementary Table 3), while the remainder appeared normal (Fig. 5h). Heterozygous *pKNU:GLUC* plants were emasculated and crossed with WT pollen to assess whether FG1 abortion results from somatic activity (i.e. expression in the MMC), or transgene activity that segregates during meiosis (i.e. gametophytic activity; Supplementary Table 4). Analysis of progeny confirmed that 54% (*n* = 59) of the F1 plants carried the *pKNU:GLUC* transgene. This suggests that germline abortion is not due to expression of *pKNU:GLUC* in the FM or female gametophyte, but is consistent with *pKNU:GLUC* affecting development of the unreduced diploid MMC.

The frequency of germline abortion in *pWUS:GLUC* lines was consistently lower than *pKNU:GLUC* lines, but similar defects were detected. Approximately 25% of the *pWUS:GLUC* ovules (*n* = 1620) showed abortion at FG1 (Fig. 5j; Supplementary Table 3). Hence, the quantitative (pKNU:GLUC) and qualitative (pWUS:GLUC) differences in callose deposition detected by DAB staining and immunolabelling are accompanied by defects in female germline development.

To assess if the defects correlate with changes in tracer molecule mobility, and thus destabilisation of the MMC-NUC symplastic barrier, we utilised the fluorescent reporters described above. pAGO5:mStr^free^ localisation was similar in *pKNU:GLUC* and WT ovules, whereby the marker was unable to enter the MMC (Fig. 3e–f). Conversely, pKNU:mStr^free^ did not spread outwards from the MMC in WT or the majority of *pKNU:GLUC* ovules (Fig. 3k, l). However, in approximately 18% (*n* = 343) of *pKNU:GLUC* ovules, mStr^free^ signal was clearly detected outside of the normal *pKNU* domain, either in the chalaza and/or nucellar cells flanking the MMC (Fig. 3m–r). To quantify this, we measured the relative fluorescence intensity in the MMC and adjoining nucellar cells (Supplementary Fig. 10). The ratio of NUC:MMC fluorescence was significantly increased in the presence of *pKNU:GLUC* from (0.39 ± 0.12) to (0.52 ± 0.16). Altogether, these observations suggest that *pKNU:GLUC* expression leads to protein movement out of the MMC, and thus partially compromises the germline-nucellus symplastic block.

The small tracer molecule HPTS did not move beyond the chalaza in *pKNU:GLUC* lines, similar to WT (Fig. 4g–l). However, in *pWUS:GLUC* plants, at least 15% (*n* = 753) of the ovules reproducibly showed increased HPTS staining intensity and mobility. Intense signal was detected in the chalaza compared to WT and *pKNU:GLUC*, and extended upwards towards the base of the nucellus (Fig. 4m–r). While no signal was detected in the MMC or meiotic tetrad of WT or *pKNU:GLUC* plants, HPTS signal was weakly detected in *pWUS:GLUC* lines in the vicinity of the MMC during expansion, and became stronger during megaspore selection (Fig. 4m–r). Collectively, these results suggest that changes in callose deposition and germline viability induced by GLUC expression are accompanied by local changes in fluorescent tracer mobility.

### *pKNU:GLUC* expression leads to changes in MMC-identity and epigenetic regulatory pathways

To assess whether cell identity is altered by pKNU:GLUC expression, we examined a range of cell-type specific markers in developing ovules. Analysis of the *pWUS:GFP*^*NLS*^ and *pPIN1:PIN1-GFP* markers in *pKNU:GLUC* ovules revealed a similar expression domain to WT, suggesting there was no obvious change in epidermal or pro-vascular cell identity (Supplementary Fig. 11a–d). Similarly, the number of ovules expressing the *pKNU:YFP*^*NLS*^ marker in the MMC was unchanged in *pKNU:GLUC* plants compared to WT, suggesting that features of MMC identity are maintained (Supplementary Fig. 11e, f). We also used immunolabelling to examine H3K27me1 histone marks, since previous studies indicated that this mark is typically present in most ovule cells but not the MMC or FM^3^. Immunolabelling of thin sections from WT ovules at stage 2-II confirmed previous findings from wholemount studies, whereby labelling was detected in the nuclei of cells in the chalaza and nucellus, but was weak or undetected in the nucleus of the MMC (*n* = 3/48 ovules; 6.25%; Supplementary Fig. 12a, b). In *pKNU:GLUC* ovules, labelling was similar to WT in the chalaza and nucellus. However, labelling was notably different in the MMC whereby 45% (*n* = 37/80) of ovules showed clear immunolabelling in the MMC nucleus (Supplementary Fig. 12c). This was confirmed using samples from different laboratories and growing conditions. Taken together, these findings suggest that although cell identity appears to be generally normal in *pKNU:GLUC* ovules, a significant proportion of MMCs exhibit an abnormal “mixed” identity.

Finally, in order to assess these defects at a transcriptomic level, we conducted transcriptome (RNAseq) analysis on WT and *pKNU:GLUC* pistils (Supplementary Table 5) at the stage when callose deposition, mStr^free^ movement and histone labelling are altered. Expression of approximately 18000 genes was detected in both genotypes, and hierarchical clustering and PCA analysis clearly distinguished *pKNU:GLUC* samples from WT (Supplementary Fig. 13a–c). The overall Log_2_(fold-change) observed for most genes was generally low, almost never reaching the value of 1 or −1, consistent with localised changes occurring in the ovule. Therefore, genes showing an FDR value ≤ 0.05 were considered DEGs (Supplementary Dataset 3). Using this criterion 349 genes were upregulated in *pKNU:GLUC* relative to WT and 133 were downregulated (Supplementary Fig. 13d). These expression trends were confirmed for multiple genes using qPCR (Supplementary Fig. 13e).

GO term enrichment analysis revealed a range of biological process terms overrepresented in the *pKNU:GLUC* DEGs, including terms for meiosis, cell cycle transition regulation, DNA and RNA metabolism (Supplementary Dataset 4; Supplementary Fig. 14, 15). These terms were also found to be overrepresented in the MMC transcriptome (relative to NUC and STK; Fig. 2). Moreover, underrepresented biological process terms in the *pKNU:GLUC* DEGs appeared to be most similar to the terms enriched in the nucellus (Fig. 2), including cell wall biogenesis (e.g., xyloglucan, pectin, and oligosaccharide metabolic process), response to hormone, cell communication and the secretory pathway (Supplementary Fig. 14, 15). Further analysis of the cellular component terms revealed that genes encoding proteins involved in chromatin remodelling were overrepresented in the DEGs, and included components of the SWI/SNF (switch defective/sucrose nonfermentable) and SWR1 (SWI2/SNF2‐related 1) complexes^53,54^ (Supplementary Fig. 15). For example, *BRAHMA* (*BRM*), the hub of the SNF/SWI complex^55^, and another complex partner, *SWI3A*, were upregulated in *pKNU:GLUC* pistils (Supplementary Fig. 13f). From the *SWR1* complex we also found upregulation of *SWC4* and *ACTIN RELATED PROTEIN 9* (*ARP9*). Other upregulated genes included *LIN52A*, a member of the DREAM complex that controls cell cycle transitions^56^, *AURORA3* (*AUR3*), a kinase important for chromosome segregation^57^, *RAS ASSOCIATED WITH DIABETES PROTEIN 51C* (*RAD51C*), essential for homologous recombination during meiosis^58,59^, and *ASYNAPTIC3* (*ASY3*), required for meiotic cell cycle crossovers^60^ (Supplementary Fig. 13f). The changes in transcript abundance are consistent with deregulation of pathways involved in MMC development, such as epigenetic reprogramming via chromatin remodelling^3^ and the transition to meiosis. These differences in expression appear to have remarkably little impact on overall ovule identity (Supplementary Fig. 11a–d), but coincide with transient deficiencies in symplastic MMC isolation, MMC identity, and dramatic downstream consequences for germline development.

## Discussion

Female gametophyte formation in plants is a complex process that depends upon a single ovule cell adopting germline identity, its progression into meiosis, entry to a haploid phase, and sustained synchronised development with surrounding diploid ovule tissues to prepare for fertilisation^7^. Hence, the mechanisms underlying communication between ovule cells, and their ability to perceive or insulate themselves against signals, are of central importance for cell differentiation and reproduction. This process shares intriguing similarities with germline development in animals, in which multiple pathways protect germ cells from a somatic fate, just as somatic cells require insulation from the germline differentiation pathway^61^. One key component of germline niche development in animals is a layer of escort cells that physically insulate the germline stem cells and prevent cell-cell contact and differentiation^62,63^. It has remained unclear whether physical insulation is required for female germline development in plants.

Here we investigated the role of β−1,3-glucan (callose), a putative component of cell insulation in plant cells, during the first phase of female gametophyte development (i.e. sporogenesis). We show that heterologous cell-type specific β−1,3-glucanase (GLUC) expression induces transient changes in callose deposition in the wall of the germline precursor (MMC), and has downstream effects on the initiation of gametogenesis. A delay in callose accumulation correlates with de-regulated cell-to-cell movement of mobile molecules, and mixed identity in a proportion of MMCs. Our findings lend support to a model in which the MMC insulates itself from surrounding cells to protects its own pool of cell-type specific regulators. This supports a functional role for callose in the establishment of a cellular environment that promotes the downstream initiation of gametogenesis.

Initially, to assess molecular features of ovule cells that appear to be sensitive to interregional signalling (reviewed in Pinto et al. ^1^), we defined the transcriptome of the MMC, nucellar epidermis, and other somatic ovule cells at a stage when intercellular connectivity becomes restricted. Individual molecular programs characteristic of each cell type were identified, and these revealed more MMC- and nucellus-specific genes than previous laser microdissection datasets^4,18^. Furthermore, our datasets provide precise information to enhance the postulated expression patterns of various genes in a recently published unsupervised single cell-RNAseq analysis of the young Arabidopsis ovule^6^. Our results confirmed the expression of around 72% of genes that distinguish the germline from putative nucellus cells reported in that dataset (Supplementary Table 2).

MMC-enriched functions detected in our data include control of cell cycle, promotion of meiosis, and restriction of mitosis, which are consistent with historical observations^28,64–67^. Thus, once the MMC is expanded and punctate callose deposits are apparent, meiosis has already initiated. By comparison, the nucellus at the same stage is enriched in signalling, cell wall remodelling and symplastic transport pathways. The enrichment of cell wall genes is consistent with the dramatic changes in cell wall polysaccharide composition that accompany early ovule development, which may assist ovule growth and rapid MMC expansion^68,69^. Moreover, the enrichment of symplastic transport pathways in the nucellus supports mobile tracer experiments showing symplastic connectivity between nucellus cells, but not the MMC. This is not due to a lack of PD in the MMC wall, since TEM imaging^15^ and PDLP1a-GFP accumulation indicate PD are present, but may instead relate to differences in PD composition that influence cell:cell connectivity.

Plasmodesmata-mediated intercellular transport is influenced by the antagonistic action of GSLs and BGs, which restrict or promote symplastic movement via the synthesis or hydrolysis of callose in the PD neck region^37^. Most of the characterised GSL genes known to have a role in callose biosynthesis^70^ are abundantly expressed in the MMC relative to the nucellus. Indeed, if the relative frequency and abundance of GSL gene transcripts compared to BGs reflects differential enzymatic activity, this may provide a plausible explanation for abundant callose accumulation in the MMC. This hypothesis could potentially be tested by multi-target knockouts of the callose biosynthetic machinery in the ovule, although such an experiment would need to be highly penetrant and MMC-specific to avoid compromising critical GSL function during earlier stages of plant growth and development^71–74^. To date, genetic studies have not been able to completely remove callose from the female germline (reviewed in ref. ^75^). In our study, heterologous expression of GLUC was only able to transiently modify callose deposition in the MMC, likely reflecting a shift in the balance of synthesis to hydrolysis, and this may provide an explanation for incomplete penetrance of the FG abortion phenotype.

**Fig. 6:**
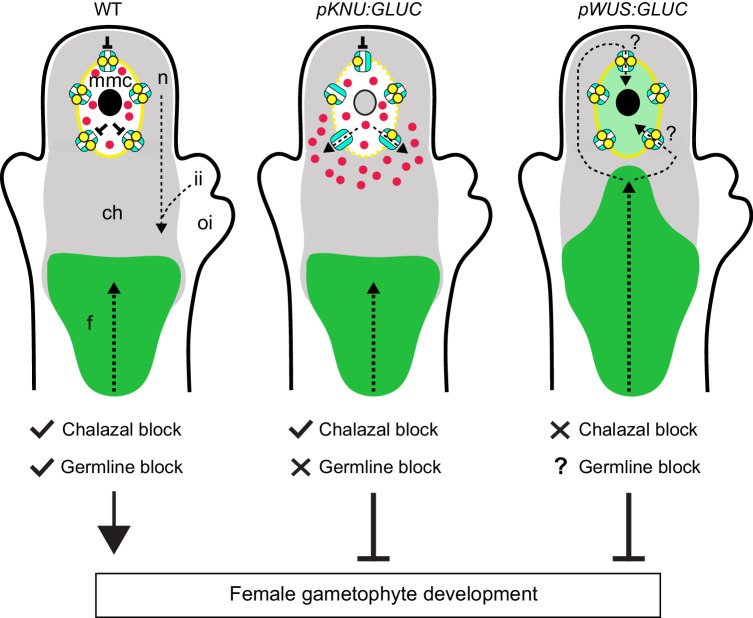
A model for the role of callose and symplastic connectivity in the young Arabidopsis ovule. Mobile molecules enter the ovule via the placenta and are unloaded into the chalaza, indicated by the zone where HPTS accumulates in wild-type (WT; green shading). Complementing this process, a distal symplastic zone allows protein movement out of the inner integument, and throughout the nucellus and chalaza, as defined by mStr^free^ spread (grey shading). Symplastic movement of mStr^free^ protein into the MMC from the nucellus or chalaza appears to be blocked in WT ovules. When GLUC is expressed under the control of *pWUS*, the symplastic block near the chalaza is eased and small molecules (e.g., HPTS) can move in an acropetal manner into the nucellus, potentially into the MMC/tetrad. It is uncertain whether this spread reflects normal movement of small molecules once they overcome the chalaza boundary. In *pKNU:GLUC* plants, delayed accumulation of callose in the MMC correlates with altered germline connectivity with surrounding cells; mobile protein (i.e. pAGO5:mStr^free^) does not appear capable of entering the MMC from the nucellus, but MMC-expressed mobile protein (pKNU:mStr^free^; red dots) spreads into the nucellus/chalaza. Coinciding with this, the MMC shows H3K27me1 labelling, which is normally removed during MMC development and is typically detected in non-germline ovule cells. The consequence of GLUC expression is abortion of gametophyte development. Arrows indicate possible routes of symplastic movement. ch chalaza, f funiculus, ii inner integument, mmc megaspore mother cell, n nucellus, oi outer integument. Drawings were created using Microsoft PowerPoint and Adobe Illustrator.

A model for symplastic isolation of the MMC (Fig. 6) proposes several non-mutually exclusive possibilities regarding the role of callose. It also highlights several missing components in terms of female gametophyte development; what is the identity of endogenous “mobile” molecules residing within or outside the MMC, and what is their function?

First, we propose that callose deposition in the MMC limits movement of regulatory molecules out of the MMC, and thereby supports the establishment of a unique germline identity. Consistent with this hypothesis, mStr^free^ is restricted to the MMC in wild-type plants, but migrates into sub-epidermal and chalazal cells adjoining the MMC in a significant proportion of *pKNU:GLUC* ovules. Although the L2 cells adjoining the MMC do not adopt MMC identity based on morphology or marker gene expression, the loss of germline-specific information (i.e. germline determinants) appears to have consequences for downstream gametophyte development. In *pKNU:GLUC* ovules, H3K27me1 chromatin immunolabelling was detected in 40% of MMCs at stage 2-II, whereas the same mark was only detected in 3% of MMCs from WT plants at the same stage. The frequency of MMCs showing H3K27me1 labelling coincides with that of ovules showing germline abortion at FG1. She et al.,^3^ reported that “repressive” marks such as H3K27me1, H3K27me3, and H3K9me1 are progressively removed from MMCs during development, potentially allowing for the establishment of a transcriptionally permissive state. The maintenance of these marks in the MMC of *pKNU:GLUC* ovules may indicate the cell remains trapped in an early phase of development, which is consistent with the up-regulation of genes characteristic of the MMC transcriptome (i.e. chromatin remodelling, meiosis, and DNA replication) in *pKNU:GLUC* pistils. Although the precise molecular basis for FG abortion after *GLUC* expression remains unknown, desynchronised MMC development relative to that of surrounding ovule tissues is an attractive hypothesis. Based on the relatively few changes revealed by RNAseq and the normal expression of ovule cell-type identity markers, FG abortion is unlikely to be caused by pleiotropic deregulation of ovule development.

A second, non-mutually exclusive possibility is that callose insulates the MMC from mobile molecules moving downwards (in a basipetal direction) from the tip of the nucellus. This transport route was highlighted in lines, where the fluorescent protein migrated from the nucellus and inner integument into the central region of the ovule. A recent study reported that small RNAs (tasiR-ARFs) follow a similar route, migrating from the nucellar epidermis into the sub-epidermal nucellus and chalaza where they restrict *ARF3* expression during MMC formation^76^. Our results are not entirely consistent with MMC callose influencing this pathway, at least for molecules >27 kDa, since mStr^free^ derived from the tip of the nucellus or inner integument was not detected in the MMC of WT ovules or lines showing altered callose accumulation.

Third, acropetal movement has also been proposed in the ovule, whereby mobile molecules (e.g. auxin and sucrose^16,22^) move upwards towards the ovule tip. In keeping with this hypothesis, Werner et al.^16^ suggested that mobile pSUC2:GFP is unloaded from the phloem and migrates throughout the young ovule, but is excluded from the MMC. A recent study also suggested that the chalaza-specific *KLUH* gene coordinates an unknown acropetal signal that moves upwards to restrict germline identity in sub-epidermal nucellar cells^65^. Using HPTS as a small (524 Da) symplastic tracer, we found that signal migrated from the phloem but only as far as the central ovule domain (chalaza) in WT and *pKNU:GLUC* plants. However, in *pWUS:GLUC* plants where the domain of GLUC expression extended further towards the centre of the ovule^69^, HPTS migrated further towards the distal end of the ovule, possibly as far as the MMC and megaspores. While our findings suggest that multiple symplastic barriers limit movement in the young ovule, further studies are required to ascertain the precise role of callose at the base of the nucellus and in the central domain of the ovule.

In all of these cases, the genes that respond to alterations in callose accumulation are of considerable interest for understanding gametophyte development, and may represent diverse pathways^1^. As described above, *pKNU:GLUC* altered the expression of multiple components of the chromatin remodelling SWI/SNF2 and SWR1 complexes, known to impact female germline cell fate and hinder its development^3,64^. These complexes destabilise DNA-histone interactions to shape chromatin architecture, thus governing the epigenetic and transcriptional landscape of cells^53,77,78^. Progression from MMC to gametophytic cell fate comprises epigenetic reprogramming, and meiosis is defined by a transient increase in general H2A.Z occupancy^3^. Future interrogation of this scaffold, particularly in terms of epigenetic regulatory molecules and their intercelluar mobility, may allow additional components of germline identity and its regulation to be revealed.

## Methods

### Plant material and growth conditions

All materials used in this study were generated in the *Arabidopsis thaliana* Columbia ecotype. WT seeds were obtained from the Nottingham Arabidopsis Stock Centre (NASC). Prior to sowing, seeds were incubated in the dark, at 4 ^o^C for 3 days. Seeds were sown on Murashige and Skoog (MS) medium [2.3 gL^−1^ MS basal salts (Duchefa Biochemie), 1% (w/v) sucrose, 1X Gamborg vitamins, 2.3 mM MES, 3 gL^−1^ gellan gum (Wako), pH 5.7] and placed in a growth incubator for 15 days. Seedlings were subsequently transferred to soil and plants were grown in growth chambers at 22 °C under long day conditions (16 h/8 h light/dark). The marker lines *pKNU:YFP*^*NLS*^, *pWUS:WUS-GFP*^18,79^, *pPIN1:PIN1-GFP*^80^, and *pSTK:STK-GFP*^19^ were described previously. For the *pKNU:GLUC* and *pWUS:GLUC* constructs, >10 primary transgenic lines were identified for each after selection on BASTA. All plants were phenotyped in the T1 and T2 generations to confirm phenotypes. Multiple lines were characterised in detail (*pKNU:GLUC* line #1, #2, #6 and *pWUS:GLUC* line #1, #4, #6) and showed similar phenotypes in terms of female abortion to those reported here. *pKNU:GLUC* line #1 and *pWUS:GLUC* line #6 were chosen for detailed analysis in the T3 generation.

For crosses, flowers from female parents were emasculated 2 days prior to anthesis and cross-pollinated. *Nicotiana benthamiana* plants were grown at 22 °C in a glasshouse under natural light.

### Protoplast isolation and collection

For each collection, around 4 flowers at developmental stage 10 (according to Schmidt et al. ^4^) were harvested. Pistils were isolated under an Olympus MVX10 MacroView stereomicroscope (Olympus) using 27-gauge hypodermic needles. A drop of enzyme solution [1 mM CaCl_2_, 0.2% (w/v) bovine serum albumin (BSA, Sigma-Aldrich), 0.2 M D-mannitol, 1% (w/v) cellulase “Onozuka” RS (Yakult), 0.5% (w/v) macerozyme R-10 (Yakult)] was added to the pistils. Using the hypodermic needles, ovules were released into the solution and major pistil debris were removed. The sample was incubated in a dark humid chamber for 30 minutes, with 85 rpm horizontal shaking at room temperature, and then placed on an Olympus IX73 inverted microscope (Olympus) equipped with a pico-pipetting system (PicoPipet, Nepagene). A G-1 glass capillary (Narishige) inserted in the PicoPipet was prepared using the micropipette puller P-97 (Sutter Instrument Co.). To stop the enzyme reaction, most protoplasts were harvested within 30 min using the PicoPipet and placed on a drop of pre-enzyme solution [1 mM CaCl_2_, 0.2% (w/v) BSA, 0.2 M D-mannitol]. Using a short exposure time of UV light, around 10 to 25 fluorescent protoplasts of each marker line were collected into 20 µL of lysis buffer supplied in the DynaBeads® mRNA DIRECT™ Micro kit (Life Technologies), snap-frozen in liquid nitrogen and stored at −80 ^o^C until mRNA extraction. A total of three biological replicates were obtained for each marker line. Each biological replicate consisted of two independent protoplast collections to allow a total of 20 to 40 cells per biological replicate. RNA sequencing was performed at the Centre for Gene Research (Nagoya University).

### RNA sequencing

Protoplast RNA extraction was performed using the DynaBeads® mRNA DIRECT™ kit Micro (Life Technologies) according to the manufacturer’s instructions with minor changes: after thawing, lysis buffer was added to a final volume of 50 µL per sample and the recommended quantities of the following reagents were reduced by half.

Directly after mRNA isolation, cDNA was synthesised and amplified using the Ovation® RNA-seq system V2 (NuGEN), as indicated in the instruction manual. Amplified cDNA was purified using the MiniElute Reaction Cleanup Kit (QIAGEN) and stored at −20 ^o^C. cDNA libraries were prepared using the TruSeq® RNA Sample Preparation V2 (Illumina) kit. The double strand cDNA was sheared in an S220 Focused-ultrasonicator (Covaris) and cDNA libraries were obtained following the recommendations in the Low Sample protocol.

The cDNA libraries were prepared using NextSeq 500/550 High Output Kit v2.5 (75 Cycles) and sequenced on a NextSeq 500 (Illumina), generating around 30 Gb of single-end 90 bp reads for each library. Raw data was deposited in the SRA archive with reference [PRJNA1077594].

For the pistil RNA sequencing experiments, three biological replicates were obtained for pKNU:GLUC and WT. Each biological replicate was a collection of 30-40 pistils from flowers at stage 10 (according to Smyth et al. ^81^) containing pre-meiotic ovules at stage 2-I to 2-III (according to Schneitz et al. ^5^), and collected from around 10 to 15 plants. Pistils were dissected under a stereomicroscope using sterile hypodermic needles and immediately snap-frozen in a DWK Life Sciences Kimble™ BioMasherII™ Closed System Micro Tissue Homogenizer (1.5 mL microcentrifuge tube) kept at −80 ^o^C in liquid nitrogen. Pistils were grinded still frozen and total RNA was extracted using the RNeasy Mini Kit (Qiagen) and eluted in 30 mL of RNAse-free water. RNA purity and concentration was measured using a NanoDrop™ One/OneC Microvolume UV-Vis Spectrophotometer (Thermo Scientific). To remove gDNA, RNA was treated with TURBO DNA-free™ Kit (Invitrogen). RNA integrity was then evaluated by verifying the presence of the 25S and 18S ribosomal RNA bands in a 1% (w/v) agarose gel.

cDNA library preparation and sequencing were performed at Novogene Co., Ltd (Hong Kong). The cDNA library (poly A enrichment) was prepared with NEBNext® Ultra™ RNA Library Prep Kit for Illumina® (NEB) and sequenced on an Illumina NovaSeq 6000 platform generating 6 Gb of raw data (150 bp, paired-end, 20 million reads) per sample. Raw data was deposited in the SRA archive with reference [PRJNA1079523].

### RNA-seq data analysis and DEG identification

The protoplast RNAseq data were pre-processed through the Galaxy server^82^ (https://usegalaxy.org/). The raw read quality was analysed using FastQC (https://usegalaxy.org/root?tool_id=toolshed.g2.bx.psu.edu/repos/devteam/fastqc/fastqc/0.72+galaxy1) with the following parameters: sliding window: 4:20; illumina clip: overrepresented sequences; min len: 50 and leading: 30 with Phred <30. Reads were pre-processed using trimomatic^83^ (https://usegalaxy.org/root?tool_id=toolshed.g2.bx.psu.edu/repos/pjbriggs/trimmomatic/trimmomatic/0.38.0) to trim low quality ends and adaptors or short reads.

The raw reads obtained for the pistil RNAseq data were pre-processed by Novogene to remove reads containing adapters, reads containing *N* > 10% (*N* represents an undetermined base), reads containing low quality (Qphred ≤ 5) base which is over 50% of the total base.

Remaining processing was done using the Discovery Environment from the Cyverse server^84^ (https://de.cyverse.org/). Trimmed reads were aligned to the Arabidopsis TAIR10 genome in STAR^85^ (app version 2.5.3a created by Chougule, 2017). The output BAM files were loaded in HT Seq-count^86^ (app version 0.6.1 created by Devisetty, 2017) to obtain read counts to genes, non-stranded, union, feature attribute: gene-id, feature type: gene. The TAIR10.45 annotation gff file downloaded on 6/11/2019 from (http://plants.ensembl.org/info/data/ftp/index.html) was used in the DEG and cluster analysis performed using SARTools^87^ (app version 3.0 (for big data) created by Devisetty, 2018) with DESeq2 method^88^, using MMC or WT as the reference condition. Loc function: median; mean-dispersion relationship: parametric; *p* adjusted value: Benjamini-Hochberg False Discovery Rate (FDR) correction, α = 0.05; outliers detection threshold (Cooks cut-off) true; independent filtering: true. PCA and clustering analysis were obtained in the DESeq2 tool and counts were transformed using the Variance Stabilizing Transformation method. Genes with a *p* adjusted value (FDR) ≤ 0.05 were regarded as differentially regulated genes.

### Gene set enrichment tests

To determine the enriched GO terms in each data set we obtained a ranked file according to Reimand et al. ^89^ that was uploaded into PANTHER^90^ and the statistical enrichment test was performed. For pKNU:GLUC RNAseq data analysis the statistically significant GO terms and associated gene number were used in REVIGO^91^ (http://revigo.irb.hr/; accessed in November 2022) to construct the semantic plots with the following options: resulting list - Large (0.9); value associated with GO term - higher absolute value is better; species – Arabidopsis thaliana (3702); semantic similarity – SimRel (default).

### mRNA in situ hybridisation

Inflorescences were fixed in 50% (v/v) ethanol, 5% (v/v) acetic acid, 0.1% (v/v) Tween 20, 4% (w/v) paraformaldehyde, embedded in paraffin wax and then sectioned to 8µm^46^. To make probes, PCR fragments from genes of interest were amplified from WT inflorescence cDNA using primers fused to the T7 promoter (see Supplementary Table 6 for primer sequences). Antisense and sense digoxigenin (DIG)-labelled probes were transcribed with T7 polymerase (ThermoFisher) and the DIG-labelling kit (Roche). For probe detection the antibody Anti-Digoxigenin-AP was used (Roche, cat. 11093274910; dilution 1:10,000). In situ hybridisation was performed using an InsituPro VSi robot (Intavis), following a standard protocol^92^.

### Transient plant transformation and analysis

Onion cells were transformed by gold particle bombardment following the protocol of Feechan et al.^93^. *Nicotiana benthamiana* plants were grown at 22 °C in a glasshouse under daylight. Approximately 3-4 leaves within the range of 3 cm to 10 cm in width were chosen for *Agrobacterium* infiltration following the protocol of Bhaskar et al.^94^. After 48 hours of growth, leaves were collected and examined by fluorescence microscopy or processed for staining. For callose staining, 0.05% w/v aniline blue in K_2_HPO_4_ was applied as described previously^43^. Images were captured using filter set 436/480 nm (callose), and punctate callose deposits were subsequently counted in a blinded experiment to assess the frequency after each treatment. Statistical differences were determined in GraphPad Prism 9 using one-way ANOVA. All transient transformation experiments were repeated at least three times.

### GLUC expression and enzyme assays

The GLUC gene was cloned into the pET151 vector, using the following primers: 5′-CACC ATG ACA TTT GCA TTT GCA TCC −3’ (forward) and 5’-CTA ATC CGA CGG ATT TAC CTT CCC GGT TAT GTT −3’ (reverse). Plasmids verified by sequencing were transformed into *E. coli* BL21 (DE3) cells (Life Technologies) by heat shock (incubation at 42 °C for 30 s). Colonies were cultivated in 10 mL overnight cultures, and 1 mL was used to inoculate a 100 mL culture, all in selective LB media containing kanamycin (50 µg mL^−1^). Cells were grown at 37 °C with shaking (180 rpm), to an OD_600_ of 0.4-0.6. Recombinant protein production was induced by the addition of 0.2 mM IPTG (isopropyl-β-D-thiogalactopyranoside) and the temperature was lowered to 30 °C for an additional 6 h. Cells were harvested by centrifugation at 4,000 g for 10 min and resuspended in 10 mL buffer A (20 mM sodium phosphate pH 7.4, 500 mM sodium chloride, 20 mM imidazole) prior to cell lysis by sonication. Soluble proteins were collected by centrifugation at 27,000 g for 30 min. The same procedure was applied to cells transformed with an empty vector. Protein concentration in the cell-free supernatant liquids was determined using the Bradford assay^95^, then increased by reducing the total volume using Amicon Ultra centrifugal filters (Millipore).

The substrates cellohexaose, laminarihexaose, tamarind xyloglucan, wheat arabinoxylan, konjac glucomannan, barley β-glucan and lichenan were purchased from Megazyme (Ireland). Laminarin was purchased from Sigma Aldrich, and curdlan was obtained from Waco Chemicals (Richmond, VA, USA). (1,3)-β-Glucans from the yeast *Saccharomyces cerevisiae* were isolated from freeze-dried cells. Cell walls prepared as described in Mélida et al.^96^ were suspended and incubated for 12 h at 4 °C in a 10% NaOH aqueous solution containing 5% urea. The sample was centrifuged for 10 min at 5000 g and the pH of the supernatant containing the alkali-soluble cell wall polysaccharides was adjusted to pH 7 with acetic acid. The solution was dialyzed against distilled water and polysaccharides were precipitated in 66% ethanol prior to lyophilisation. The fraction essentially consisted of a mixture of (1,3)- and (1,6)-β-glucans. For assays of enzyme activity, substrates (5 g L^−1^) was incubated with the recombinant protein (0.2 g L^−1^) in a total reaction volume of 500 µL in 50 mM sodium citrate buffer, pH 6.5. Reaction mixtures were incubated overnight (~16 h) at 30 °C with agitation (rotary shaking at 180 rpm). Reactions were performed using proteins extracted from cells expressing the GH17 enzyme and cells harbouring a control plasmid. Control reactions were also performed without the addition of any protein. All assays were performed in triplicate.

To measure the concentration of reducing sugars in the assay, an equal volume (500 µL) of 3,5-dinitrosalicylic acid (DNSA) reagent was added to the reaction, which was heated to 95 °C for 10 min, prior to cooling to room temperature, and the measurement of absorbance at 575 nm (Miller 1959). Absorbance was measured using a Cary 50 UV/Vis spectrophotometer (Varian). Absorbance measurements were compared to those from a standard curve of glucose (0.01–0.1 g L^−1^) glucose to determine the concentration of released reducing sugars. The absorbance values from control reactions were taken as a measure of background activity in the *E. coli* culture filtrates and subtracted from those of the GH17 assays.

### Protein structure predictions and phylogenetic analysis

Amino acid sequences were analysed using InterProScan (https://www.ebi.ac.uk/interpro/result/InterProScan) and subcellular location was predicted using DeepLoc 2.0 (https://services.healthtech.dtu.dk/services/DeepLoc-2.0/), using default parameters in both cases.

Sequences of GLUC-like proteins were aligned using Clustal Omega^97^. Substitution model selection was performed using ModelTest-NG^98^, with LG-GAMMA(4) selected as the best-fit model. The best-known maximum likelihood tree was constructed using RAxML-NG v1.2.0^99^. ML searches were performed on 100 random and 100 parsimony trees with the final tree chosen according to highest GAMMA-based likelihood. The number of bootstrap trees calculated were determined using the RAxML autoMRE criterion, with convergence achieved at 900 replicates.

### Immunolabelling of cell wall epitopes

Individual Arabidopsis flowers were fixed overnight at 4 ^o^C in 0.25% (w/v) glutaraldehyde, 4% (w/v) paraformaldehyde, 4% (w/v) sucrose in phosphate buffered saline (PBS) [0.8% (w/v) NaCl, 0.02% (w/v) KCl, 0.144% (w/v) Na_2_HPO_4_, 0.024% (w/v) KH_2_PO_4_, pH 7.4] overnight at 4 °C. Samples were embedded in LR White as described previously^100^, and 1 µm sections were obtained with a Leica EM UC6 microtome. The sections were placed on glass slides and used for immunolocalisation with monoclonal antibodies including LM20^101^, JIM13^102^ (1:100 dilution; Kerafast, Boston, MA, USA) and Anti-callose BS400-2^103^ (1:100 dilution; Biosupplies, Bundoora, Vic., Australia). AlexaFluor® 555 anti-rat IgG (Invitrogen A-21434) and AlexaFluor® 488 anti-mouse IgG (Invitrogen A-11001) were used as secondary antibodies (1:200 dilution). The protocol for immunohistology is described in Burton et al.^104^. The slides were mounted in 90% (v/v) glycerol for observation. The immunolabelling experiment was carried out at least three times on serial sections of different pistils to verify differences in labelling.

### Immunodetection of H3K27me1

Immunodetection of H3K27me1 was performed following a previous method with modifications^105^. Tissues were fixed in FAA, embedded, and sectioned as described in^106^. After dewaxing and rehydration, paraffin sections (6 μm) were microwave-heated in 10 mM citrate buffer (pH 6.0) for 3 min at high power for antigen retrieval. Sections were incubated with blocking buffer [3% (m/v) BSA in PBS buffer] for 1 h before overnight incubation with primary antibodies for H3K27me1 (1:400 dilution, ThermoFisher Scientific) at 4 °C in a humidified chamber. AlexaFluor® 488 conjugated anti-rabbit IgG (1:400 dilution, Invitrogen) was used as secondary antibody to visualise immunosignals. Sections were counterstained in 5 μg/ml PI, rinsed in water and imaged by a Zeiss M2 AxioImager (GFP filter set 470 nm/525 nm, PI filter set 545 nm/605 nm).

### Construct generation

The full length GLUC sequence was amplified from *Hieracium piloselloides* D36 cDNA^46^ using the following primers GLUC_FWD 5’-ATGACATTTGCATTTGCATCCTT and GLUC_REV 5’-CTAATCCGACGGATTTACCTTCCCGGTT and cloned into pCR4-TOPO. Details of the constructs generated in this study including* pKNU:GLUC*, *pWUS:GLUC*, *p35S:GLUC-GFP*, *p35S:GFP-GLUC*, *pKNU:mStrawb*^*free*^ *pAGO5:mStrawb*^*free*^ *pAGO5:PDLP1a-GFP* and *pKNU:PDLP1a-GFP* are available upon request.

### Quantitative PCR

To confirm expression level of GLUC RNAseq deregulated genes, RNA was isolated in triplicates from pistils stage 10 (according to Smyth et al.^81^) similarly to the RNAseq sample collection. About 350 ng of RNA from each sample was treated with DNAse I, RNase-free (Thermo Scientific™, USA) and cDNA was synthesised using Maxima First Strand cDNA Synthesis Kit for RT-qPCR (Thermo Scientific™) and oligo(dT)20 primers to initiate the reactions.

*SAMDC* (*AT3G02470*), *SAC52* (*AT1G14320*) and *YLS8* (*AT5G08290*) were chosen as reference genes, as these were highly and stably expressed across all samples of the protoplast RNAseq data.

Primers for genes of interest were designed using Primer3 v.4.1.0^107,108^ (Supplementary Table 6; Thermo Scientific^TM^). cDNA from each sample were used for relative gene expression, calculated using the 2-ΔΔCt method^109^. Data were statistically treated using GraphPad Prism 9 software. For each analysis, relative expression differences were compared using unpaired two-sided student’s t-test. Statistical significance was considered α = 0.05. The raw data underlying the averages of gene expression are shown in the Source Data file.

### Preparation of plant material for microscopy

For ovule clearing, fluorescence analysis and decolourised aniline blue staining, ovules were dissected from flowers as per Tucker et al.^18^ and staged according to Schneitz et al.^5^. Whole ovules were cleared using Hoyer’s solution (160 g of chloral hydrate, 100 mL of water, and 50 mL of glycerol). For callose staining, 0.05% (w/v) aniline blue in K_2_HPO_4_ was applied to fresh pistils and ovules as described in Levy et al.^43^. For marker line analysis, *pKNU:PDLP1a*-*GFP* reporter lines, mobility assays, fertility assessment and callose staining and immunolabelling, whole or sectioned ovules were observed using an Axio Imager M2 microscope (Zeiss) equipped with DIC prism for bright field images, UV light for fluorescence detection and filters for GFP (filter set 470 nm/525 nm), RFP/mStrawberry (filter set 545 nm/605 nm), YFP (filter set 500 nm/535 nm) and CFP (filter set 436 nm/480 nm). Images were captured with an AxioCamMR R3 camera (Zeiss), using Zen 2 pro software (Zeiss). To enable comparisons between ovules, DIC and fluorescence data in each experiment was collected using the same settings and exposure times. Any adjustments to brightness/contrast were performed uniformly across all samples (using batch analysis) before being compiled in Adobe Photoshop and Illustrator.

For confocal microscopy, ovules were observed under the Nikon A1R Laser Scanning Confocal microscope equipped with GaAsP PMT detectors, and a 60x objective (Plan Apo VC 60xA WI). GFP excitation was performed with a 488 nm argon laser at 6% power with gain set between 65 and 120. Fluorescence was detected at 525–550 nm. No averaging or accumulation was applied. Z-stacks were acquired in unidirectional mode, 12 bits resolution and digital zoom was set to 1. The NIS-Elements C control software (version 5.02.03; Nikon) was used for image acquisition. Maximum intensity projections were obtained using FIJI (z-projection tool).

### Tracer dye assays

2.5 mM of 8-hydroxyprene-1,3,6-trisulfonic acid trisodium salt (HPTS) was prepared in H_2_O and applied to plants by soaking excised inflorescence stems in an Eppendorf tube for 3 h. Flowers were then removed and dissected on microscope slides to reveal the ovules prior to analysis using a M2 AxioImager (Zeiss).

### Statistics and reproducibility

Apart from the RNAseq data analysis, the significance of differences between groups was tested within Prism 9 (GraphPad Software Inc.). Statistical analysis was performed using two-tailed unpaired Student’s t test or one-way ANOVA to determine differences between two groups. *p*-values or *f*-values < 0.05 were interpreted as statistically significant. Data are presented as mean ± Standard Error of Mean (SEM) or ± Standard Deviation (SD) depending on the experiment. Other details such as the number of replicates and the level of significance are indicated in the text, figure legends and/or Supplementary Data. The representative figures shown in the paper were selected from at least three independent experiments, unless otherwise stated.

### Reporting summary

Further information on research design is available in the Nature Portfolio Reporting Summary linked to this article.

### Supplementary information

Supplementary information

Reporting Summary

Description of Additional Supplementary Files

Supplementary Dataset 1

Supplementary Dataset 2

Supplementary Dataset 3

Supplementary Dataset 4

### Source data

Source data

## Data Availability

The raw RNAseq data generated in this study have been deposited in the sequence read archive (SRA) repository of NCBI under BioProject accessions: PRJNA1077594 [https://www.ncbi.nlm.nih.gov/bioproject/1077594] and PRJNA1079523 [http://www.ncbi.nlm.nih.gov/bioproject/1079523]. Additional details regarding protocols and datasets used in this study are available from the corresponding author. Source data are provided with this paper.
