# Supplementary material for: Germline β−1,3-glucan deposits are required for female gametogenesis in Arabidopsis thaliana
Source: Nat Commun. 2024 Jul 12;15:5875. doi: 10.1038/s41467-024-50143-0 (PMC11245613; doi:10.1038/s41467-024-50143-0)
Supplement: Supplementary file 1 — Supplementary information [file 41467_2024_50143_MOESM1_ESM.pdf]

Supplementary Data for Pinto et al.,

"Germline β-1,3-glucan deposits are required for female gametogenesis in *Arabidopsis thaliana*"

Supplementary Figures

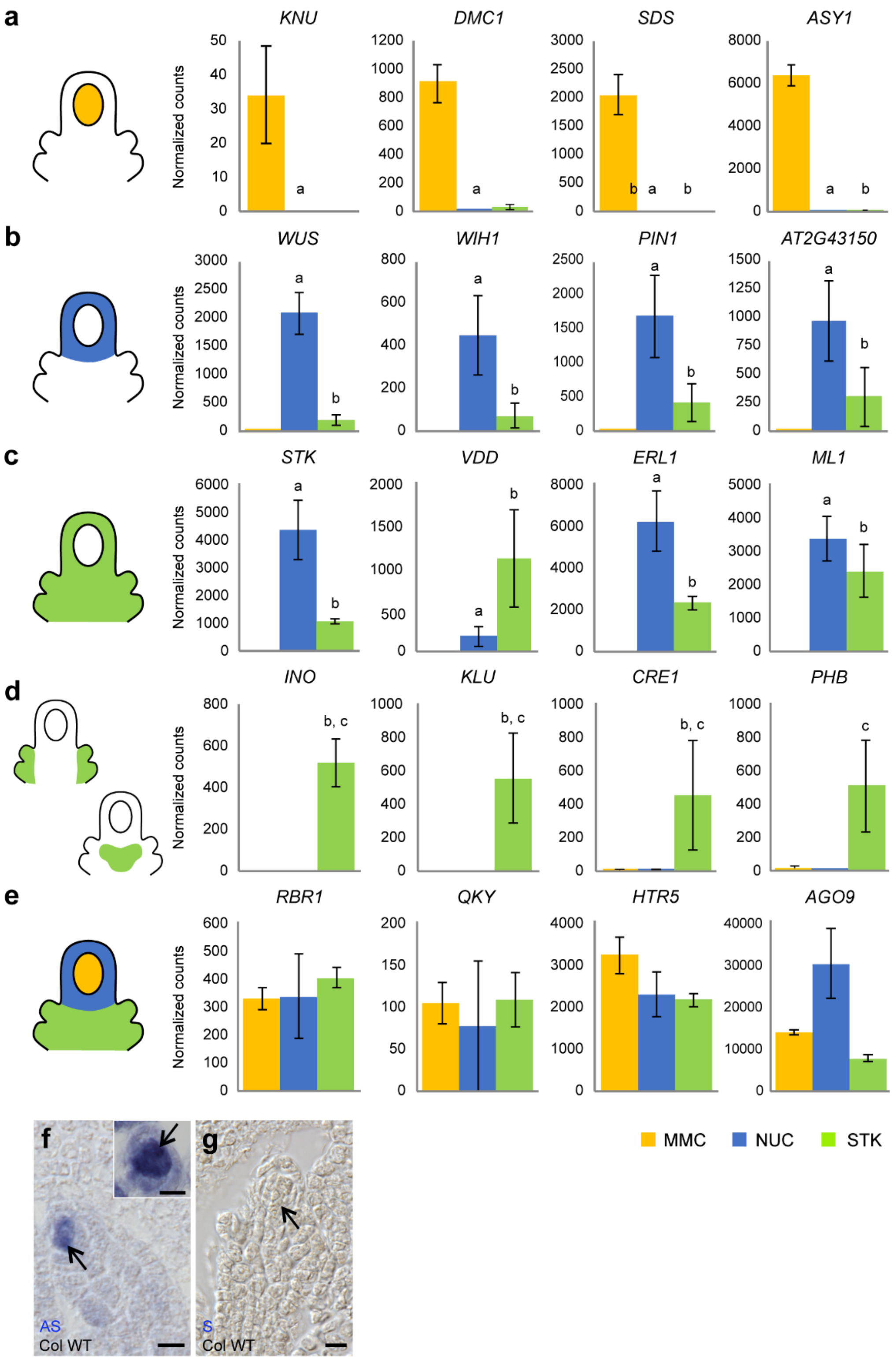

Supplementary Fig. 1 – Expression of genes in different ovule cell types based on cell-type specific transcriptional profiling. (a) Genes with MMC-restricted expression. (b) Previously published NUC-enriched genes. (c) Genes showing a similar expression

profile to STK. (d) Genes expressed in a restricted somatic domain, such as the integument primordia or chalaza. (e) Genes with expression detected in many ovule cell types. Expression level is represented as average normalized read counts from three biological replicates. Bars represent (±) standard error. Letters on graphs: a – FDR ≤ 0.05 between NUC and MMC; b – FDR ≤ 0.05 between STK and MMC; c – FDR ≤ 0.05 between STK and NUC. *AGO9 – ARGONAUTE 9; ASY1 – ASYNAPTIC 1; CRE1 – CYTOKININ RESPONSE 1; DMC1 – DISRUPTION OF MEIOTIC CONTROL 1*; *ERL1 – ERECTA-LIKE 1*; *HTR5 – HISTONE THREE RELATED 5*; *INO – INNER NO OUTER*; *KLU – KLUH; KNU – KNUCKLES*; *ML1 – MERISTEM LAYER 1*; *PHB – PHABULOSA*; *PIN1 - PIN-FORMED 1*; *QKY – QUIRKY*; *RBR1 – RETINOBLASTOMA-RELATED 1; SDS – SOLO DANCERS*; *STK – SEEDSTICK*; *VDD – VERDANDI*; *WIH1 – WINDHOSE 1; WUS – WUSCHEL*. (f – g) *In situ* hybridization with anti-sense (AS) and sense (S) probe for *ASY1*. Inset is a cross section of the ovule through the MMC. This experiment was conducted three times using pistils from independent plants. Arrows point the MMC. Scale bars = 10 μm. Drawings were created using Microsoft PowerPoint and Adobe Illustrator.

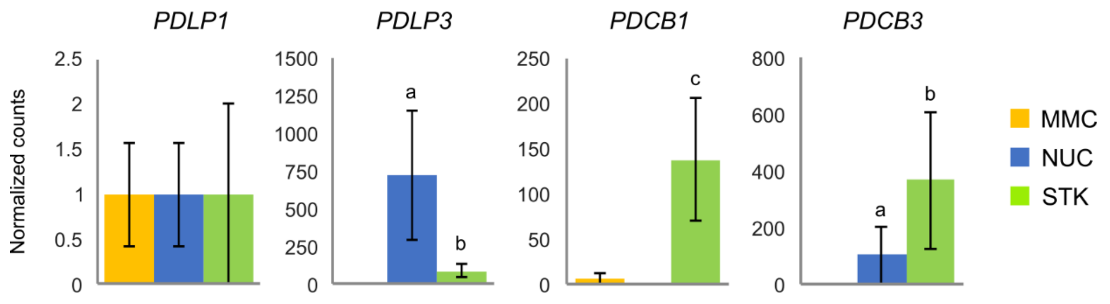

Supplementary Fig. 2 – Expression of genes involved in regulation of plasmodesmal permeability. *PLASMODESMATA-LOCALIZED PROTEIN 1* (*PDLP1*) is not expressed in the ovule. *PDLP3* expression is confined to the somatic cells, mainly the nucellus. *PLASMODESMATA CALLOSE BINDING PROTEIN 1* (*PDCB1*) and *PDCB3* are more abundant in the STK sample. Expression level is represented as average normalized read counts from three biological replicates. Bars represent (±) standard error. Letters on graphs: a – FDR ≤ 0.05 between NUC and MMC; b - FDR ≤ 0.05 between STK and MMC; c - FDR ≤ 0.05 between STK and NUC.

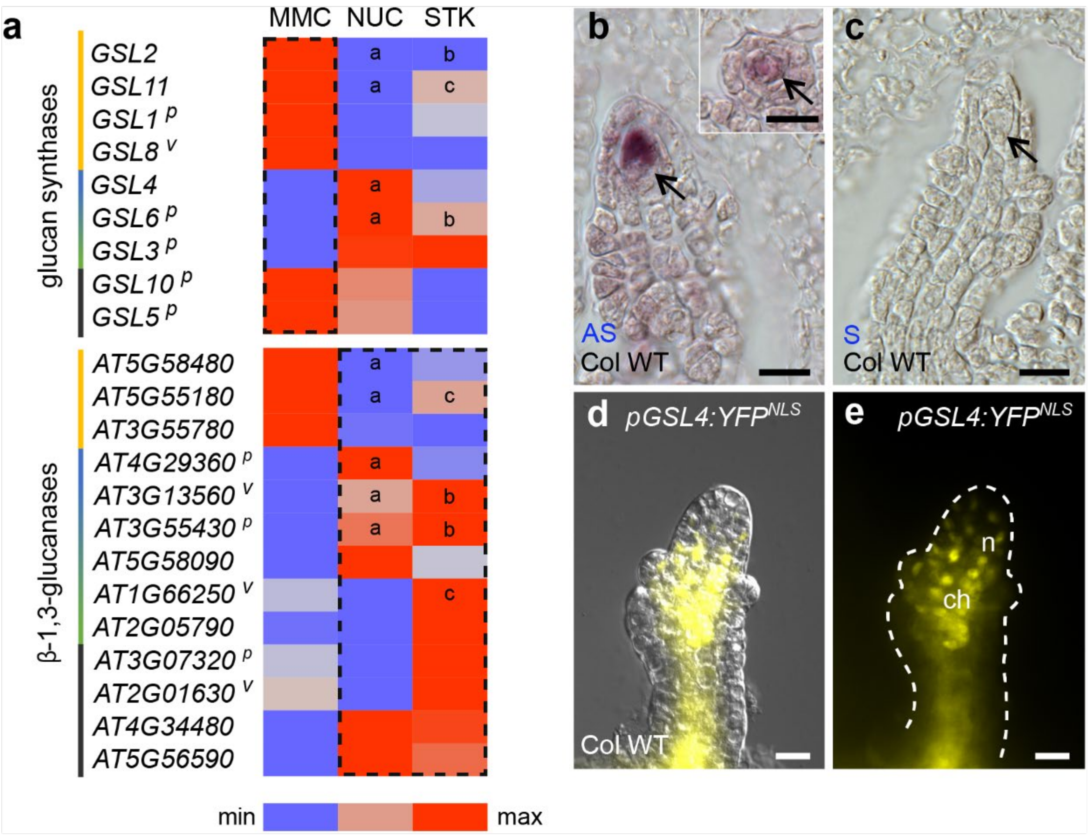

Supplementary Fig. 3 – Expression of *GLUCAN SYNTHASE-LIKE* (*GSL*) and β-1,3-glucanase genes putatively involved in plasmodesmal callose accumulation. (a) Heat map of average normalized read counts detected in the MMC, somatic cells (NUC) or whole ovule (STK). Expression represents the average of three biological replicates for each sample type. Letters on heatmap: a – FDR ≤ 0.05 between NUC and MMC; b - FDR ≤ 0.05 between STK and MMC; c - FDR ≤ 0.05 between STK and NUC. Annotations next to gene name: v - indicates that corresponding protein localization in PDs has been validated; p - indicates that corresponding protein localization in PDs is predicted (according to[1]). Dashed black line notes higher (absolute) representation of synthases or glucanases in the MMC or other cell types. (b, c) Expression of *GSL2* via *in situ* hybridization. Anti-sense (AS) probe validates *GSL2* mRNA presence specifically in the MMC (arrow). Inset is a cross-section of an ovule through the MMC.

This experiment was conducted three times using pistils from different plants. (d, e) *pGSL4:YFP*^{NLS} expression confirms that *pGSL4* drives expression in NUC. (d) Merged DIC and YFP fluorescence microscopy. (e) Fluorescence microscopy. ch – chalaza; n – nucellus. Scale bars = 10 μm.

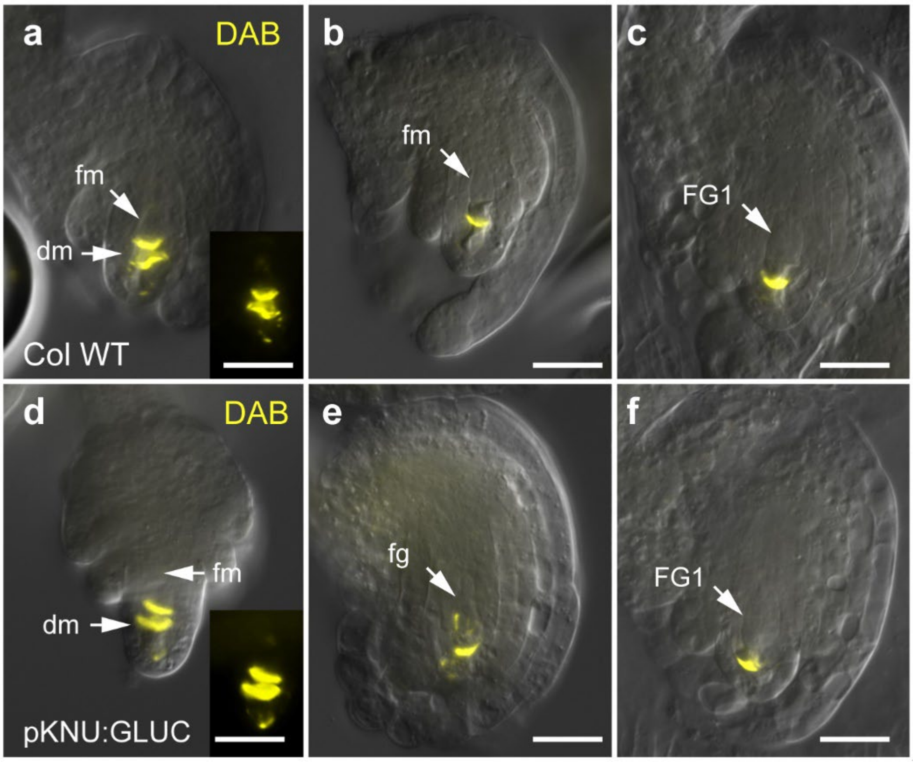

Supplementary Fig. 4 – Callose deposition in ovules expressing pKNU:GLUC, from tetrad to FG1 stage. Decolourised Aniline Blue (DAB) stains callose (yellow) in the cell plates separating degenerating megaspores (dm) and functional megaspore (fm) in wild-type (WT; a) and pKNU:GLUC (d). When the fm or the one-nucleate female gametophyte (FG1) is established, DAB stains callose in the micropylar region of the ovule corresponding to the degenerated megaspores both in WT (b, c) and pKNU:GLUC (e, f). Images result from merging DIC and CFP fluorescence microscopy. Insets show DAB fluorescence only. Experiments were repeated on 6 independent occasions and representative micrographs are shown. Scale bars = 20 μm.

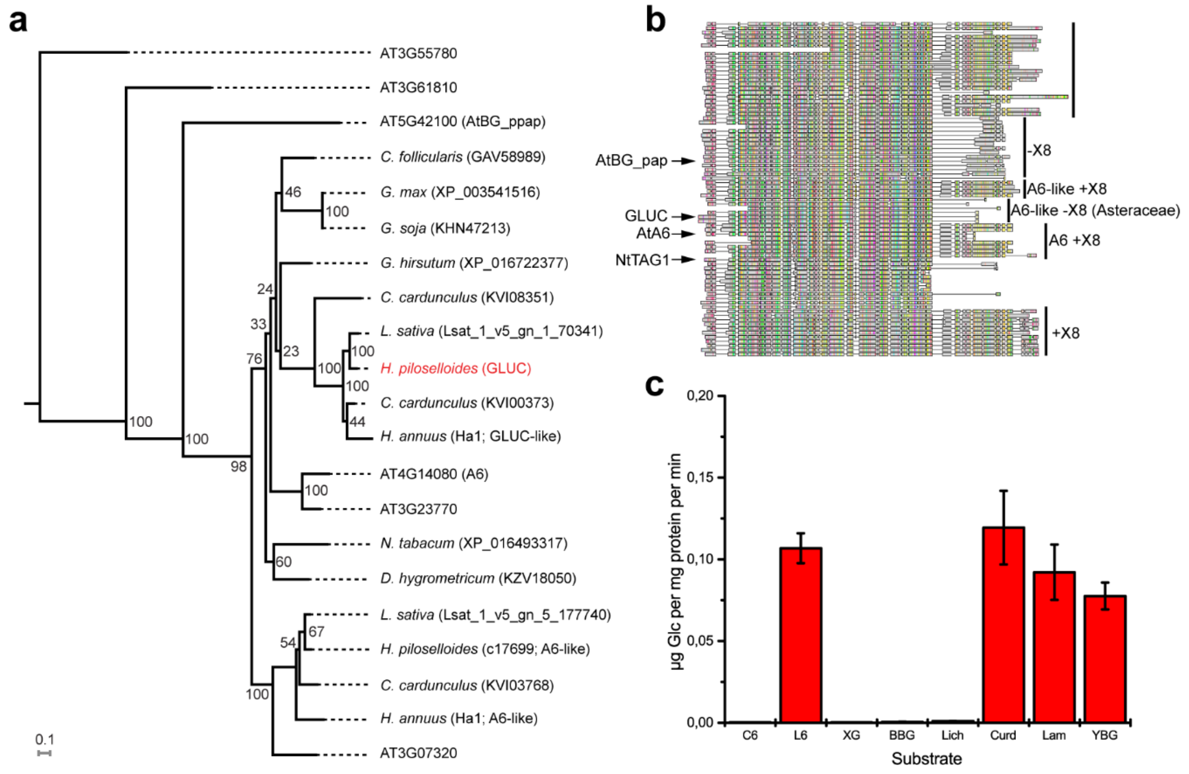

Supplementary Fig. 5. Analysis of the GLUC protein sequence. (a) Phylogenetic comparisons of A6-like β-1,3-glucanase sequences from Arabidopsis, selected Asteraceae (including GLUC in red) species and other dicotyledonous species. The best-known maximum likelihood tree was generated using RAxML-NG v1.2.0 with the LG-GAMMA(4) substitution model and 900 bootstrap iteration. (b) Protein alignments of β-1,3-glucanase sequences including GLUC. No X8 domain is detected in GLUC from *Hieracium* and several other Asteraceae species, which differs from most A6-like sequences. The previously characterized Arabidopsis AtBG_pap (plasmodesmata associated protein) also lacks an X8 domain. (c) Incubation of lysate from *E. coli* expressing GLUC releases glucose from selected substrates which is typical of β-1,3-glucanase activity. Substrate code: C6: cellohexaose, L6: laminarihexaose, XG: xyloglucan, BBG: barley β-glucan, Lich: lichenan, Curd: curdlan, Lam: laminarin, YBG: yeast β-glucan. Bars represent ± standard deviation from three replicates.

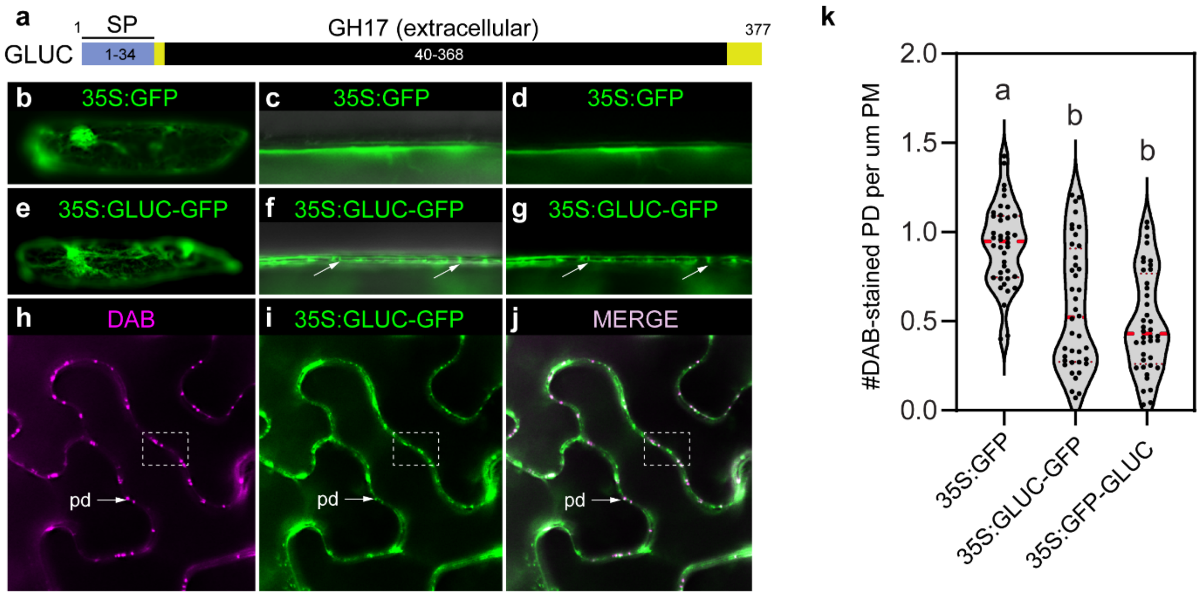

Supplementary Fig. 6 – Determination of GLUC subcellular localisation and *in planta* β-1,3-glucanase activity. (a) The predicted GLUC protein structure includes a signal peptide (SP) and an extracellular GH17 region. Numbers indicate amino acid positions. (b – d) onion cells were transiently transformed with a control 35S:GFP construct, allowing GFP detection (green) in different compartments of the cell. (e – g) onion cells were transformed with a 35S:GLUC-GFP construct, and GFP was observed in multiple compartments including punctate spots at the cell periphery (white arrows). (h – j) *N. benthamiana* cells transformed with a 35S:GLUC-GFP construct and stained with decolorized aniline blue (DAB). (h) DAB-stained callose deposits (purple) are associated with plasmodesmata (pd); (j) GLUC-GFP partially co-localises (white) with these callose deposits (see dashed square). (k) Violin plot representing the abundance of DAB-stained plasmodesmata (PD) in *N. benthamiana* epidermal cells. The number of DAB stained PD per µm of plasma membrane (PM) significantly decreased when either C- or N-terminal fusions of GLUC were transformed into *N. benthamiana* cells suggesting GLUC leads to PD callose hydrolysis. Each transient transformation was repeated four times. Letters represent

statistical differences calculated using one-way ANOVA ($p \leq 0.001$). a – i – Fluorescence microscopy; j – Merged CFP (for DAB fluorescence detection) and GFP fluorescence.

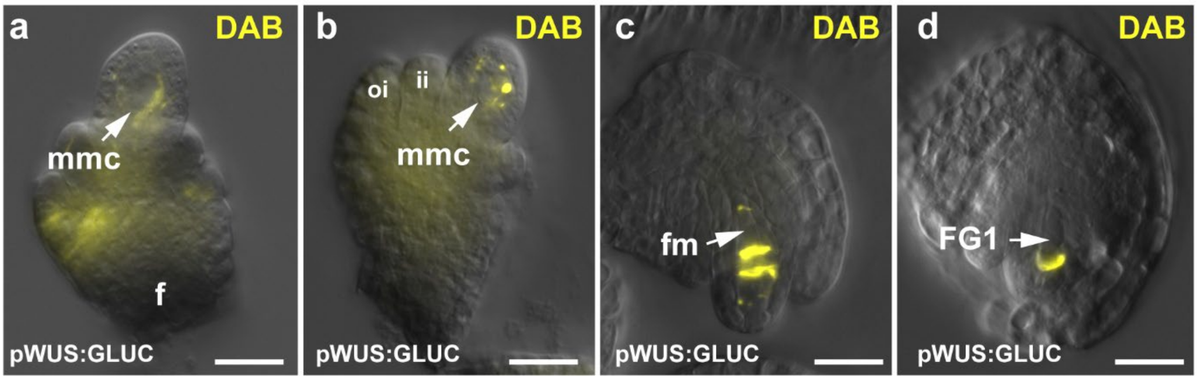

Supplementary Fig. 7 - Callose deposition in ovules expressing pWUS:GLUC. Ovules were stained with Decolorized Aniline Blue (DAB). (a) Callose staining (yellow) in pWUS:GLUC ovules was occasionally diffuse, but the majority of ovules showed a similar pattern to WT (compare with Figure 5 and Supplementary Fig. 4). (b) WT-like staining around the megaspore mother cell (mmc). (c) WT-like staining of the meiotic tetrad. (d) WT-like staining of non-selected megaspores in ovules containing a one-nucleate female gametophyte (FG1). Images result from merging DIC and CFP channels via epifluorescence microscopy. f, funiculus; fm, functional megaspore; ii, inner integument; oi, outer integument. Experiments were repeated on 6 independent occasions and representative micrographs are shown. Scale bars = 20 μm.

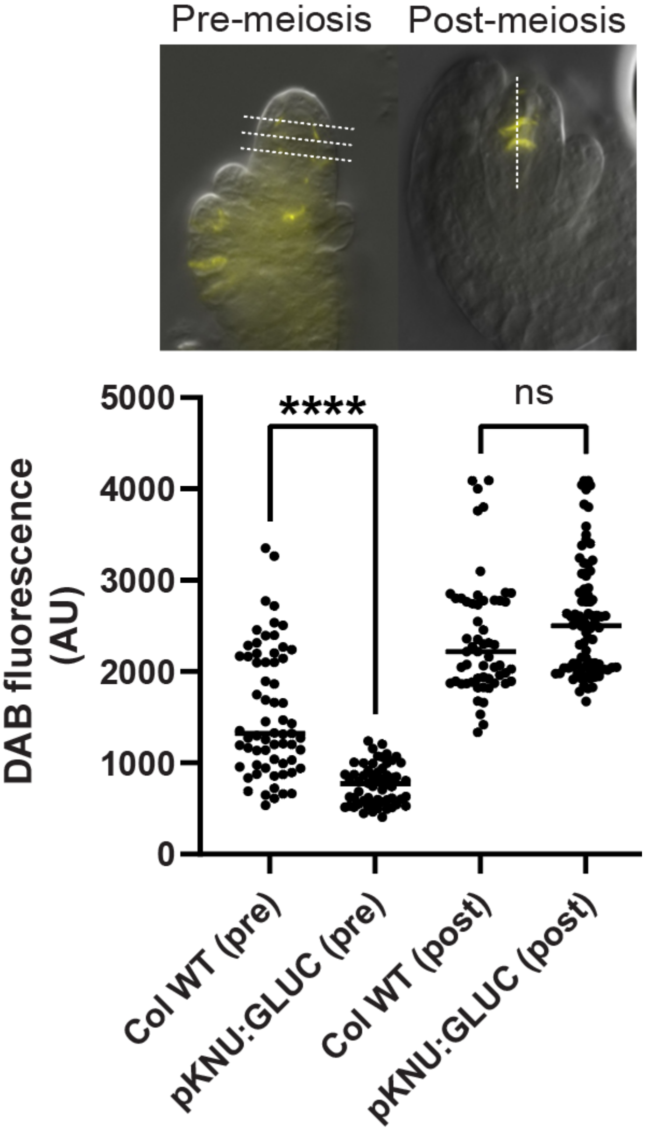

Supplementary Fig. 8 – Measurement of decolourised aniline blue (DAB) staining intensity in WT and pKNU:GLUC ovules. Data was extracted from ZEN software using the profile function (as per[2,3]). Multiple regions were examined and averaged for each MMC (pre-meiosis) while a single profile was generated for ovules containing a meiotic tetrad (post-meiosis)**.** These are indicated by the dashed lines. The maximum intensity was collected from each profile from each ovule (n = 65 (pre) and 57 (post) for Col WT, 58 (pre) and 86 (post) for pKNU:GLUC) and this always aligned with the region corresponding to the walls. Experiments were repeated on 6 independent occasions

and representative micrographs are shown Significance was assessed using a two-tailed Student's T-test. **** ≤0.0001.

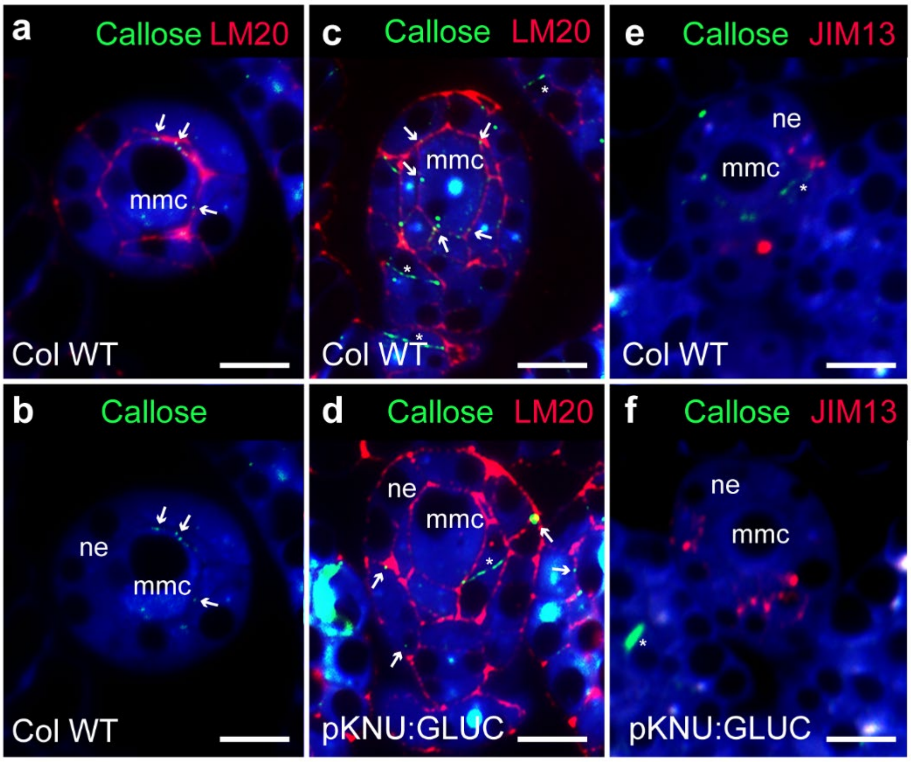

Supplementary Fig. 9 – Immunolabelling confirms that callose deposits are reduced in the MMC wall of pKNU:GLUC ovules at stage 2-II to 2-III. Thin sections of WT (a – c) and pKNU:GLUC (d) ovules were co-labelled with LM20 (pectin; red) and BS400-2 (callose; green) antibodies. Thin sections of WT (e) and pKNU:GLUC (F) ovules were also co-labelled with JIM13 (AGPs; red) and BS400-2 antibodies (green). (a – c) Punctate callose deposits were detected in the MMC wall of most WT ovules (88%, n = 78), but were only detected in 59% (n = 63) of pKNU:GLUC ovules (d – f). (c – f) No differences were observed between genotypes for the labelling with LM20 and JIM13. For all sections, DAPI-channel autofluorescence was used as a counterstain (blue signal). (a, c – f) Merged GFP (callose), dsRED (LM20 or JIM13) and DAPI (autofluorescence) channels via epifluorescence microscopy. (b) Merge of callose and autofluorescence, showing the callose deposits detected in (a). White arrows: punctate callose deposits. White asterisks show callose deposits in walls of somatic

cells. (a, b) transverse sections of the nucellus tip. (c – f) longitudinal sections of whole ovules. Immunolabelling experiments were repeated three times and representative micrographs are shown. Scale bars = 10 μm.

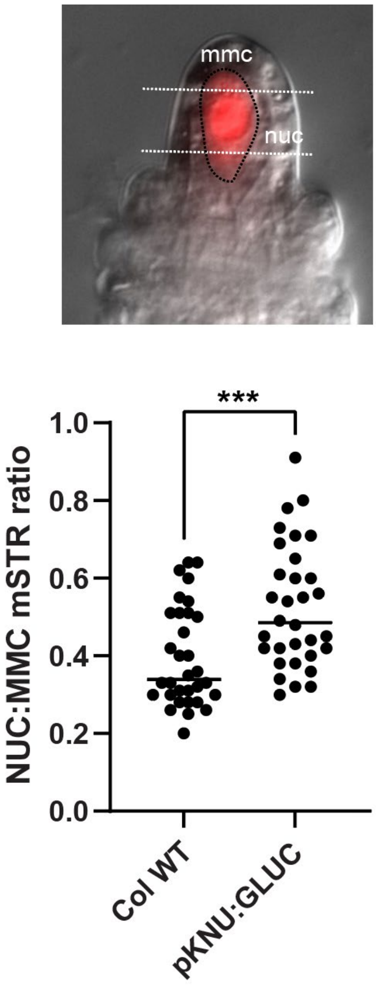

Supplementary Fig. 10 – Relative mSTR^{free} fluorescence intensity in the MMC and nucellus in Col WT and pKNU:GLUC ovules at stage 2-II to 2-III . The image shows pKNU:mSTR^{free} in an ovule from Col WT. Intensity measurements were collected as per Supplementary Figure 8, although two profiles were averaged for each MMC instead of three. The ratio was determined by comparing the fluorescence in the nucellus (region between the MMC wall and outer epidermis) to that of the MMC. This corresponds to the zone where mStr signal was typically observed in effected ovules (see Fig. 3 N, P, R). The MMC nucleus was avoided due to saturated intensity. Significance was assessed using a two-tailed Student's T-test *** ≤0.001. Experiments

were repeated on 5 occasions from 10 dissected pistils; only ovules at the depicted stage and orientation were included in the analysis. n = 32 ovules for each genotype.

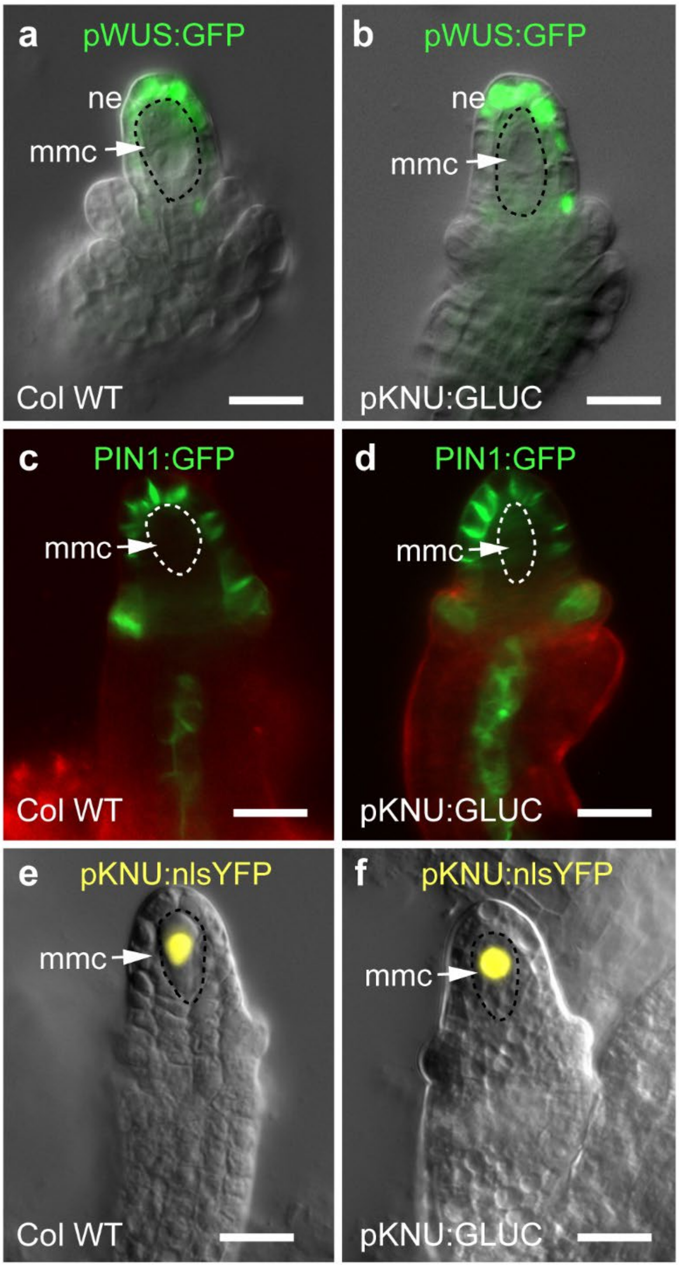

Supplemental Figure 11 – Expression of ovule marker genes in wild-type and pKNU:GLUC ovules. (a, b) The pattern of pWUS:GFP^{NLS} in the ovule appears similar between wild-type and pKNU:GLUC. (c, d) The pattern of PIN1-GFP in the ovule appears similar between wild-type and pKNU:GLUC. (e, f) pKNU:YFP^{NLS} expression in the MMC is indistinguishable between wild-type and pKNU:GLUC. mmc, megaspore mother cell; ne, nucellar epidermis. Experiments were repeated on 3 occasions with

independent F3 progeny of crosses and representative micrographs are shown. Scale bars = 15 μm.

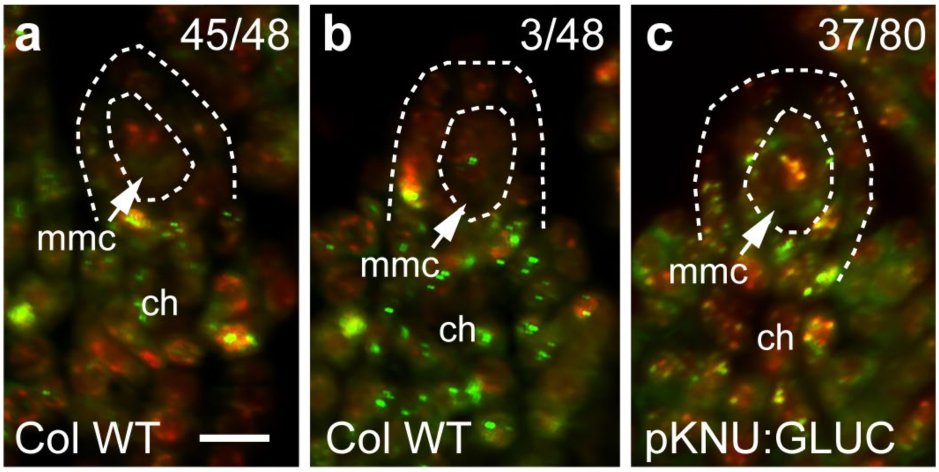

Supplementary Fig. 12 – Immunodetection of H3K27me1 in sections of Arabidopsis ovules at stage 2-II to 2-III. Green signal highlights H3K27me1 labelling, red indicates counterstaining with propidium iodide, yellow indicates overlapping signal. Dashed lines are used to outline the mmc and the nucellus. (a) Col WT ovule showing a MMC with no H3K27me1 labelling in the MMC nucleus. Signal is detected in surrounding ovule cells, and a similar pattern was observed in 45/48 ovules containing an MMC. (b) Col WT ovule showing H3K27me1 labelling in the MMC nucleus. This pattern was observed in 3/48 ovules. (c) pKNU:GLUC ovule showing accumulation of H3K27me1 labelling in the MMC nucleus. A similar pattern was observed in 37/80 ovules, while the remaining 43 could not be distinguised from WT (a). ch, chalaza; mmc, megaspore mother cell. Immunolabelling experiments were repeated on 3 independent occasions and on multiple floral buds. Scale bar = 10 μm and images are shown to scale.

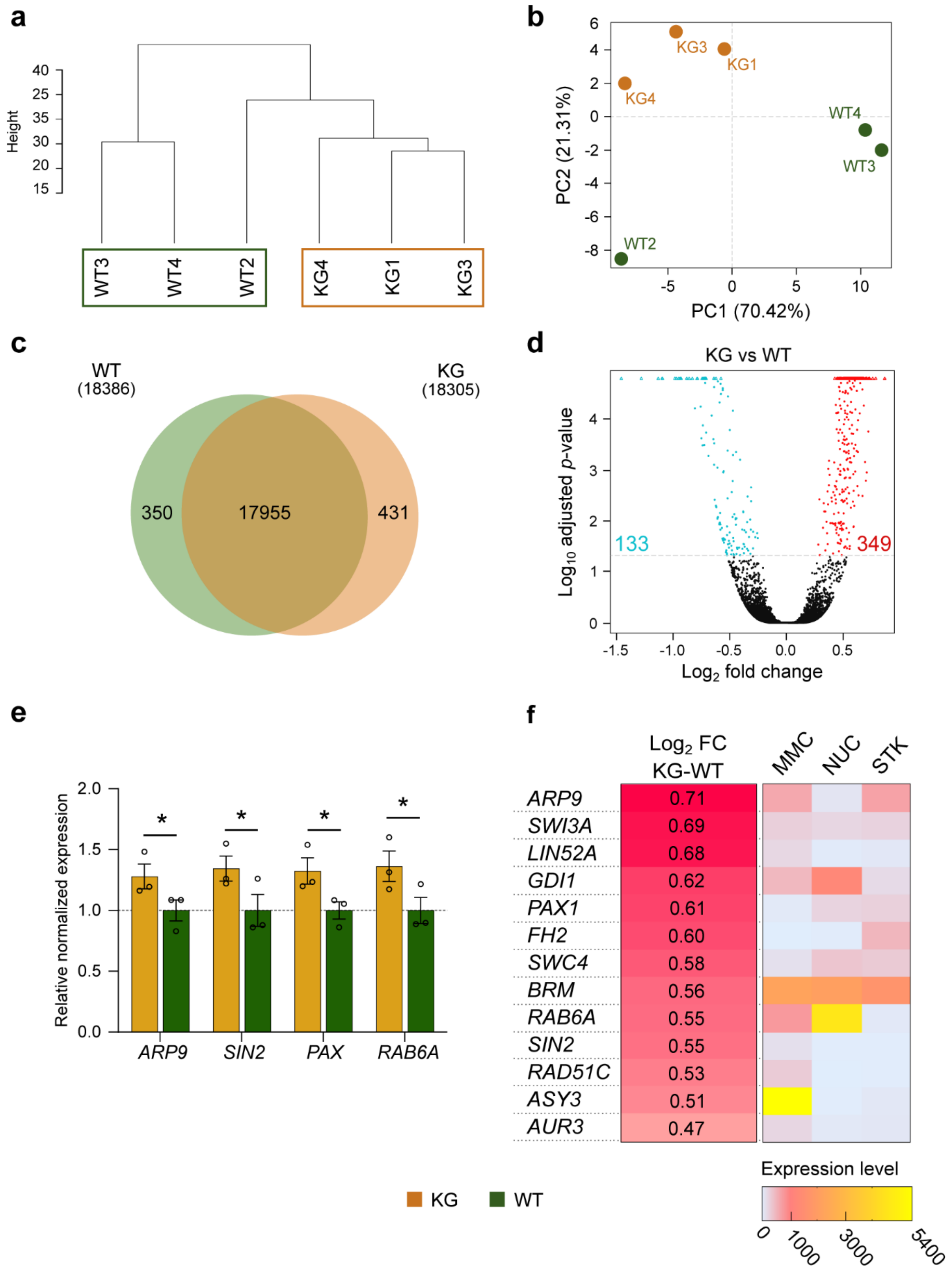

Supplementary Fig. 13 – RNAseq of *pKNU:GLUC* pistils and differential expression of genes of interest. RNAseq quality assessment: (a) Hierarchical clustering

dendrogram and (b) Principal Component Analysis (PCA) scatter plot. (c) Venn diagram showing the number of genes expressed in each genotype (in parenthesis) and the overlap between transcriptomes. (d) Volcano plot depicting the differentially expressed genes (blue: downregulated genes = FDR < 0.05; red: upregulated genes = FDR < 0.05). y axis – adjusted p-value is the FDR calculated as indicated in the methods section. (e) qPCR analysis of target genes in *pKNU:GLUC* (KG) and wild type (WT). Data are shown as mean normalized expression relative to WT. Grey line marks the WT threshold. Error bar: +/- SEM. Asterisks note statistically significant differences tested by unpaired two-tailed Student's t-test (∗ ≤ 0.05). Each gene represents an independent experiment, therefore statistical tests were done per target gene. Golden, *pKNU:GLUC* samples (KG). Dark green, wild type (WT). WT2, WT3, WT4 = WT biological replicates; KG1, KG3, KG4 = KG biological replicates. (f) Selected differentially expressed genes in the *KG* RNAseq. In the first column, the Log_2 (fold change) of selected meiotic and chromatin genes differentially expressed in the *KG* RNAseq is presented. The heatmap on the right shows the expression levels of those genes in the protoplast RNAseq data. MMC - transcript level in *pKNU:YFP*^{NLS} samples. NUC - transcript level in *pWUS:GFP-WUS* samples. STK - transcript level in *pSTK:STK:GFP* samples. Expression level for the protoplast RNAseq is represented as normalized counts (as described for Supplementary Fig.1).

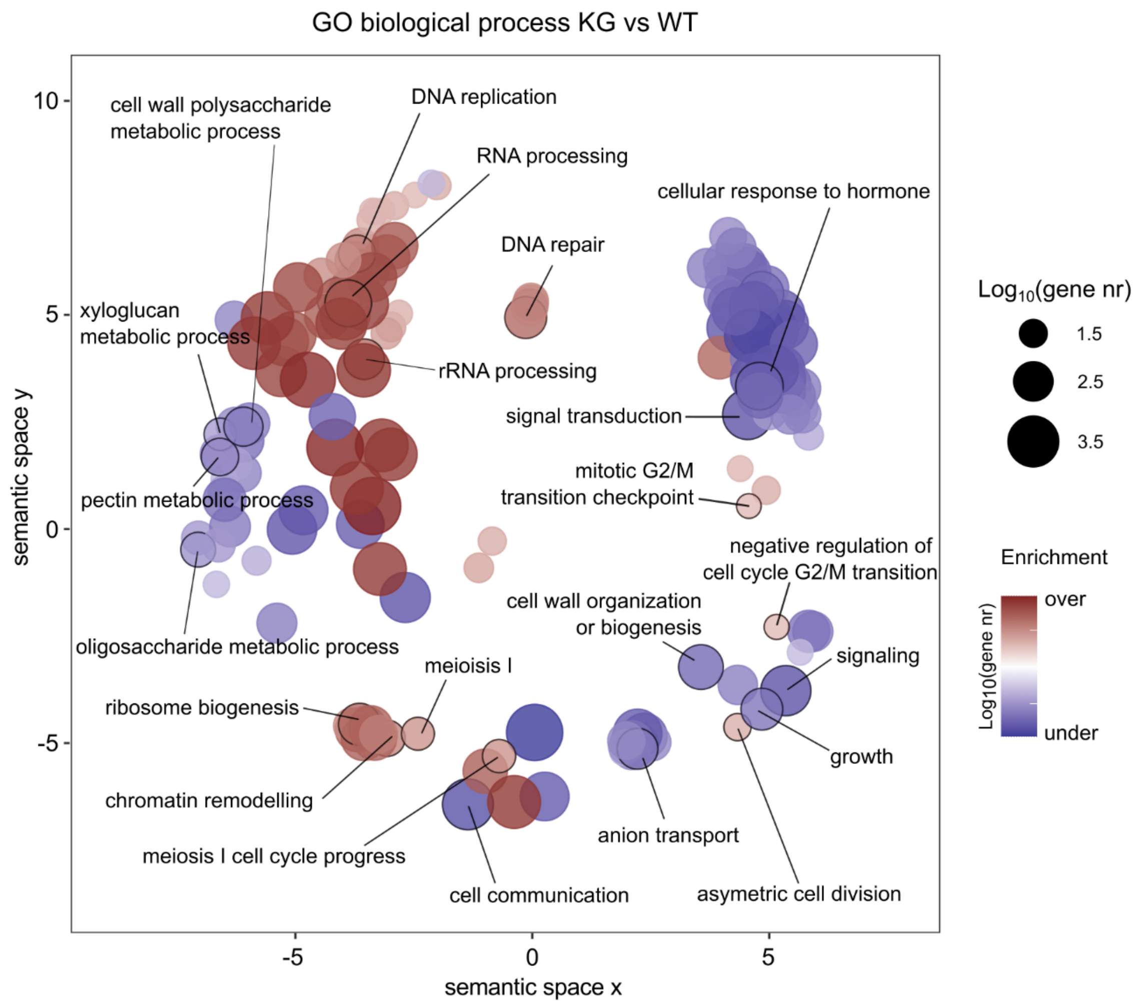

Supplementary Fig. 14 – Semantic plot of GO biological terms for pKNU:GLUC (KG) vs. WT transcriptome comparison. Each GO term is represented by a circle, where the size is proportional to the log10 of the gene number that each GO term contains. The colours of the circles relate to enrichment; red are overrepresented and blue are underrepresented. The log10 of gene number was used to create the colour gradient. GO terms with a higher number of genes have a darker colour. The semantic position was calculated using REVIGO[4] with 156 GO terms. See Supplementary Data 3 for the list of GO terms obtained using PANTHER.

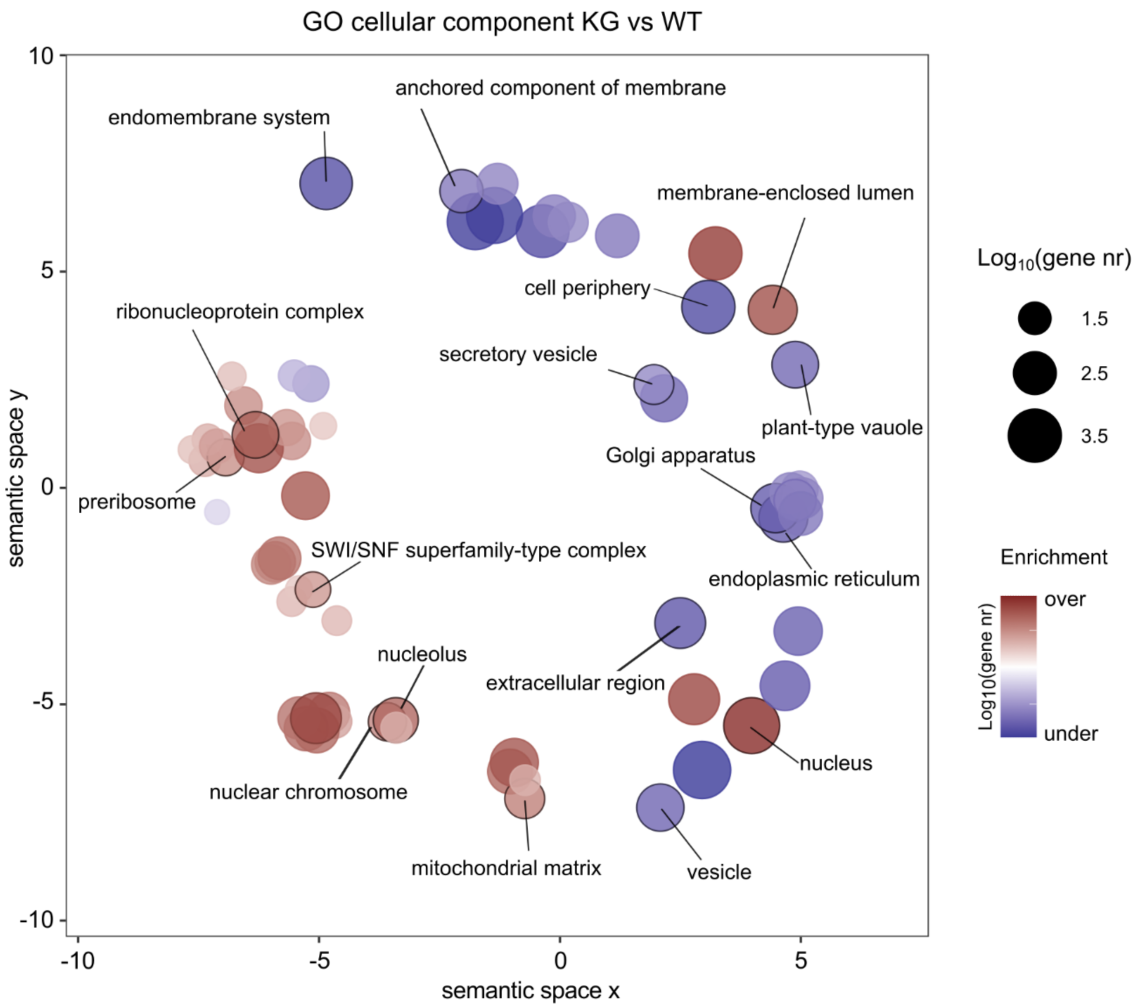

Supplementary Fig. 15 – Semantic plot of GO cellular component terms for pKNU:GLUC (KG) vs. WT transcriptome comparison. Each GO term is represented by a circle, where the size is proportional to the log10 of the gene number that each GO term contains. The colours of the circles relate to enrichment, in red are overrepresented, and blue are underrepresented. The log10 of gene number was used to create the colour gradient. GO terms with a higher number of genes have a darker colour. The semantic position was calculated using REVIGO[4] with 65 GO terms. See Supplementary Data 3 for the list of GO terms obtained using PANTHER.

Supplementary Tables

Supplementary Table 1 - Read number and coverage obtained for each biological replicate used in the cell-type specific transcriptomes. M – million reads.

	Sample	Total reads	% Mapped reads	% Uniquely mapped reads
pKNU	M35	4.9 M	94.2	86.7
	M46	4.4 M	95.2	88.6
	M79	3.6 M	94.0	83.7
pWUS	N05	12.5 M	73.6	70.3
	N26	14.9 M	77.3	73.7
	N39	9.7 M	80.1	75.9
pSTK	S35	6.1 M	88.5	81.8
	S47	4.9 M	89.5	81.5
	S68	6.1 M	85.6	81.2

Supplementary Table 2 - Direct comparison between scRNAseq data published by Hou et al., 2021 and transcriptomes provided in this study. Feature genes correspond to the genes that are expressed in the MMC or nucellus epidermis subclusters (as described in Hou et al., 2021). "# genes showing the same trend" refers to the overlap between the subcluster genes and those upregulated in the MMC or NUC/STK solely based on Log_2FC (fold change). For the "# differentially expressed genes (DEG) showing the same trend", the upregulation is statistically significant in the cell-type-specific MMC or NUC/STK transcriptomes.

		**# of feature genes in subclusters**	**# genes showing same trend**	**% Feature genes**	**# DEGs showing same trend**	**% Feature genes**
MMC subclusters	In AC1.1	101	34	*33.7*	2	*2.0*
	In MMC1.10.3	74	67	*90.5*	8	*10.8*
	In MMC2.6.3	341	292	***85.6***	59	***17.3***
Nucellus epidermis subclusters	In AC1.1	101	74	*73.3*	26	*25.7*
	In MMC1.10.1 & MMC1.10.2	71	56	*78.9*	24	*33.8*
	In MMC2.6.1 & MMC2.6.4	177	130	***73.5***	50	***28.3***

Supplementary Table 3 – Abortion phenotype observed for each genotype studied. Numbers represent the average of three independent transgenic lines ± standard deviation. Mature ovules were cleared in Hoyer's solution for phenotyping. n = total number of ovules analysed.

**Genotype**	Percentage FG1 abortion ± standard deviation	n
pKNU:GLUC	39.2 ± 10.2	3288
pWUS:GLUC	25.5 ± 5.2	1620
Col WT	1.3 ± 1.4	3335
pKNU:PDLP1a:GFP	1.0 ± 1.6	495

Supplementary Table 4 – Analysis of GLUC transgene transmission in reciprocal crosses. A two-sided Fisher exact test was used to determine that the GLUC phenotypes are due to sporophytic effects of the transgene (α = 0.05). n = number of ovules analysed. n.s. – not statistically significant. Hz – heterozygous. Wt – wild-type.

**Cross** ♀ x ♂	n	Expected percentage GLUC progeny	Observed percentage of GLUC progeny	*p*-value (Fisher exact test)
pKNU:GLUC (Hz) x WT	59	50	54. 2	n.s.
WT x pKNU:GLUC (Hz)	33	50	36. 4	n.s.
pWUS:GLUC (Hz) x WT	17	50	64.7	n.s.
WT x pWUS:GLUC (Hz)	23	50	60.9	n.s.

Supplementary Table 5 - Read number and coverage obtained for each biological replicate used in the pistil transcriptomes. M – million reads.

	Sample	Total read number	% Mapped reads	% Uniquely mapped reads
WT	WT2	25.2 M	98.67	96.91
	WT3	23.1 M	98.60	96.50
	WT4	21.8 M	98.61	96.39
pKNU:GLUC	KG1	22.0 M	98.93	96.81
	KG3	20.4 M	98.93	97.26
	KG4	20.4 M	98.94	96.71

Supplementary Table 6 – List of primers used in this study

Name	Gene	5' -> 3' sequence	Notes
OSP_093	AT2G13680	CAGACAAGGCTTCTCTTCAA	GSL2 gDNA fragment
OSP_094	AT2G13680	GAGTGCTAAAGAGTGACGAT	GSL2 gDNA fragment
OSP_095n	AT2G13680	TAATACGACTCACTATAGCAGACAAGGCTTCTCTTCAA	GSL2 in situ probe primer (with T7 adaptor)
OSP_096n	AT2G13680	TAATACGACTCACTATAGGAGTGCTAAAGAGTGACGAT	GSL2 in situ probe primer (with T7 adaptor)
OSP_077	AT1G67370	AGAGCTTTGCGGTACATGGT	ASY1 gDNA fragment
OSP_078	AT1G67370	TGGTGTCTGATAGAGGTGGTG	ASY1 gDNA fragment
OSP_079n	AT1G67370	TAATACGACTCACTATAGAGAGCTTTGCGGTACATGGT	ASY1 in situ probe primer (with T7 adaptor)
OSP_080n	AT1G67370	TAATACGACTCACTATAGTGGTGTCTGATAGAGGTGGTG	ASY1 in situ probe primer (with T7 adaptor)
1911_F	AT2G17950	GCGATGCTTATCTGGAACATC	WUS qPCR primer
1911_R	AT2G17950	AACTTCCGATTGGCCATACTT	WUS qPCR primer
2061_F	AT1G73590	GACACTCCCCAACACTCTAG	PIN1 qPCR primer
2061_R	AT1G73590	AGCTTAGCTCCACGGTACTC	PIN1 qPCR primer
2038_F	AT5G21150	AGGAATCACCACACCTGGAG	AGO9 qPCR primer
2038_R	AT5G21150	CCCACAAAAACCGACAGAAT	AGO9 qPCR primer
2040_F	AT2G27880	ACCCATCAGGGAGCTAAGGTTC	AGO5 qPCR primer
2040_R	AT2G27880	TAGACGGGTCTTGTGTCACTCC	AGO5 qPCR primer
oMT524	HpGLUC	AGATCTATGGCTCTTGTCTTCCTCTCTTTC	GLUC genotyping primer
oMT525	HpGLUC	AGATCTCTAATCCGACGGATTTACCTTC	GLUC genotyping primer
1581_ARP9_qPCR_F	AT5G43500	CTCAGATTGGTGGTGCTGGT	ARP9 qPCR primer
1582_ARP9_qPCR_R	AT5G43500	ACGAATTGTGGCTCCGTTC	ARP9 qPCR primer
1595_SIN2_qPCR_F	AT2G41670	CTACGGTTGGTCCATTGCCT	SIN2 qPCR primer
1596_SIN2_qPCR_R	AT2G41670	CAATCCGCTCTTCACCCACT	SIN2 qPCR primer
1587_PAX_qPCR_F	AT2G44830	CCCGAGTCACCAGCAACTAG	PAX qPCR primer
1588_PAX_qPCR_R	AT2G44830	CGGTTCGGTCTCCATCTGTC	PAX qPCR primer
1591_RAB6A_qPCR_F	AT2G44610	TCGAACGGTCAGACTTCAGT	RAB6A qPCR primer
1592_RAB6A_qPCR_R	AT2G44610	TCCCACAAGCACGACTATGAC	RAB6A qPCR primer
1160_SAMDC_fw	AT3G02470	TTGGTAAGTACTGTGGATCGCC	SAMDC reference qPCR primer
1161_SAMDC_rv	AT3G02470	CTGCTAGATTCCCTCGTCCTTC	SAMDC reference qPCR primer

1152_SAC52_fw	AT1G14320	CGTCGTGCTAAGTTCAAGTTCC	SAC52 reference qPCR primer
1153_SAC52_rv	AT1G14320	CTTCTCTTGCCTCAACTTGGTG	SAC52 reference qPCR primer
1154_YLS8_fw	AT5G08290	AAGATCAACTGGGCTCTCAAGG	YLS8 reference qPCR primer
1155_YLS8_rv	AT5G08290	TGGGAAGCTCGATTAGTAACGG	YLS8 reference qPCR primer

Supplementary References

1. Kirk P, Amsbury S, German L, Gaudioso-Pedraza R, Benitez-Alfonso Y. A comparative meta-proteomic pipeline for the identification of plasmodesmata proteins and regulatory conditions in diverse plant species. *BMC Biol* **20**, 128 (2022).
2. Chowdhury J, Henderson M, Schweizer P, Burton RA, Fincher GB, Little A. Differential accumulation of callose, arabinoxylan and cellulose in nonpenetrated versus penetrated papillae on leaves of barley infected with Blumeria graminis f. sp. hordei. *New Phytol* **204**, 650-660 (2014).
3. Aditya J*, et al.* The dynamics of cereal cyst nematode infection differ between susceptible and resistant barley cultivars and lead to changes in (1,3;1,4)-beta-glucan levels and HvCslF gene transcript abundance. *New Phytol* **207**, 135-147 (2015).
4. Supek F, Bosnjak M, Skunca N, Smuc T. REVIGO summarizes and visualizes long lists of gene ontology terms. *PLoS One* **6**, e21800 (2011).
