# Supplementary material for: Germline β−1,3-glucan deposits are required for female gametogenesis in Arabidopsis thaliana
Source: Nat Commun. 2024 Jul 12;15:5875. doi: 10.1038/s41467-024-50143-0 (PMC11245613; doi:10.1038/s41467-024-50143-0)
Supplement: Supplementary file 2 — Reporting Summary [file 41467_2024_50143_MOESM2_ESM.pdf]

nature portfolio

Corresponding author(s): *Double-anonymous peer review submissions: write DAPR and your manuscript number here instead of author names.*

Last updated by author(s): *YYYY-MM-DD*

Reporting Summary

Nature Portfolio wishes to improve the reproducibility of the work that we publish. This form provides structure for consistency and transparency in reporting. For further information on Nature Portfolio policies, see our Editorial Policies and the Editorial Policy Checklist.

Statistics

For all statistical analyses, confirm that the following items are present in the figure legend, table legend, main text, or Methods section.

n/a	Confirmed	
☐	☒	The exact sample size (*n*) for each experimental group/condition, given as a discrete number and unit of measurement
☐	☒	A statement on whether measurements were taken from distinct samples or whether the same sample was measured repeatedly
☐	☒	The statistical test(s) used AND whether they are one- or two-sided<br>*Only common tests should be described solely by name; describe more complex techniques in the Methods section.*
☒	☐	A description of all covariates tested
☒	☐	A description of any assumptions or corrections, such as tests of normality and adjustment for multiple comparisons
☐	☒	A full description of the statistical parameters including central tendency (e.g. means) or other basic estimates (e.g. regression coefficient) AND variation (e.g. standard deviation) or associated estimates of uncertainty (e.g. confidence intervals)
☐	☒	For null hypothesis testing, the test statistic (e.g. *F*, *t*, *r*) with confidence intervals, effect sizes, degrees of freedom and *P* value noted<br>*Give P values as exact values whenever suitable.*
☒	☐	For Bayesian analysis, information on the choice of priors and Markov chain Monte Carlo settings
☒	☐	For hierarchical and complex designs, identification of the appropriate level for tests and full reporting of outcomes
☒	☐	Estimates of effect sizes (e.g. Cohen's *d*, Pearson's *r*), indicating how they were calculated

Our web collection on statistics for biologists contains articles on many of the points above.

Software and code

Policy information about availability of computer code

Data collection	Images of in situ hybridization and resin section were collected using NIS-Elements AR (Nikon, version v4.2);<br>Images of LM19 immunolabelling were collected using ZEN blue edition (Zeiss, v3.5);<br>Confocal images were collected with NIS-Elements AR (Nikon, v4.30.01);

Data analysis	qRT-PCR data were analyzed with QuantStudio (Thermo Fisher, v1.3).<br>ZEN blue edition (Zeiss, v3.5) was used for fluorescence signal quantification.<br>For RNA-seq: The protoplast RNAseq data were pre-processed through the Galaxy server82 (https://usegalaxy.org/). The raw read quality was analysed using FastQC (https://usegalaxy.org/root?tool_id=toolshed.g2.bx.psu.edu/repos/devteam/fastqc/fastqc/0.72+galaxy1). Reads were pre-processed using trimomatic83 (https://usegalaxy.org/root?tool_id=toolshed.g2.bx.psu.edu/repos/pjbriggs/trimmomatic/trimmomatic/0.38.0) to trim low quality ends and adaptors or short reads. emaining processing was done using the Discovery Environment from the Cyverse server84 (https://de.cyverse.org/). Trimmed reads were aligned to the Arabidopsis TAIR10 genome in STAR85 (app version 2.5.3a created by Chougule, 2017). The output BAM files were loaded in HT Seq-count86 (app version 0.6.1 created by Devisetty, 2017) to obtain read counts to genes, non-stranded, union, feature attribute: gene-id, feature type: gene. The TAIR10.45 annotation gff file downloaded on 6/11/2019 from (http://plants.ensembl.org/info/data/ftp/index.html) was used in the DEG and cluster analysis performed using SARTools87 (app version 3.0 (for big data) created by Devisetty, 2018) with DESeq2 method88, using MMC or WT as the reference condition. PCA and clustering analysis were obtained in the DESeq2 tool and counts were transformed using the Variance Stabilizing Transformation method.<br>Protein analysis: Amino acid sequences were analysed using InterProScan (https://www.ebi.ac.uk/interpro/result/InterProScan) and subcellular location was predicted using DeepLoc 2.0 (https://services.healthtech.dtu.dk/services/DeepLoc-2.0/), using default parameters in both cases.<br>Graphpad Prism 9 and Microsoft Excel 2016 were used for statistical analysis and graphical data presentation.

For manuscripts utilizing custom algorithms or software that are central to the research but not yet described in published literature, software must be made available to editors and reviewers. We strongly encourage code deposition in a community repository (e.g. GitHub). See the Nature Portfolio guidelines for submitting code & software for further information.

Data

Policy information about availability of data

All manuscripts must include a data availability statement. This statement should provide the following information, where applicable:

- Accession codes, unique identifiers, or web links for publicly available datasets
- A description of any restrictions on data availability
- For clinical datasets or third party data, please ensure that the statement adheres to our policy

All data generated or analyzed during this study were included in this published article and supplementary files. Bio-reagents are available for research propose upon request from the corresponding author under a material transfer agreement.

Research involving human participants, their data, or biological material

Policy information about studies with human participants or human data. See also policy information about sex, gender (identity/presentation), and sexual orientation and race, ethnicity and racism.

Reporting on sex and gender	*Use the terms sex (biological attribute) and gender (shaped by social and cultural circumstances) carefully in order to avoid confusing both terms. Indicate if findings apply to only one sex or gender; describe whether sex and gender were considered in study design; whether sex and/or gender was determined based on self-reporting or assigned and methods used.*<br>*Provide in the source data disaggregated sex and gender data, where this information has been collected, and if consent has been obtained for sharing of individual-level data; provide overall numbers in this Reporting Summary. Please state if this information has not been collected.*<br>*Report sex- and gender-based analyses where performed, justify reasons for lack of sex- and gender-based analysis.*
Reporting on race, ethnicity, or other socially relevant groupings	*Please specify the socially constructed or socially relevant categorization variable(s) used in your manuscript and explain why they were used. Please note that such variables should not be used as proxies for other socially constructed/relevant variables (for example, race or ethnicity should not be used as a proxy for socioeconomic status).*<br>*Provide clear definitions of the relevant terms used, how they were provided (by the participants/respondents, the researchers, or third parties), and the method(s) used to classify people into the different categories (e.g. self-report, census or administrative data, social media data, etc.)*<br>*Please provide details about how you controlled for confounding variables in your analyses.*
Population characteristics	*Describe the covariate-relevant population characteristics of the human research participants (e.g. age, genotypic information, past and current diagnosis and treatment categories). If you filled out the behavioural & social sciences study design questions and have nothing to add here, write "See above."*
Recruitment	*Describe how participants were recruited. Outline any potential self-selection bias or other biases that may be present and how these are likely to impact results.*
Ethics oversight	*Identify the organization(s) that approved the study protocol.*

Note that full information on the approval of the study protocol must also be provided in the manuscript.

Field-specific reporting

Please select the one below that is the best fit for your research. If you are not sure, read the appropriate sections before making your selection.

[x] Life sciences [ ] Behavioural & social sciences [ ] Ecological, evolutionary & environmental sciences

Life sciences study design

All studies must disclose on these points even when the disclosure is negative.

Sample size	Sample sizes were determined by previous pilot experiments to be sufficient to achieve desired outcomes. Sample sizes are indicated in the Figures, legends and main text.
Data exclusions	No data were excluded from the analysis.
Replication	All experimental findings were reproduced in several independent biological experiments (n) with multiple technical replicates. The number of repeats is indicated in the figures and figure legends. The main conclusions were confirmed in different lines and in different laboratories (Adelaide and Porto). All attempts to replicate the experiments were successful and approved by multiple researchers.
Randomization	All plants materials use in this study were grown in controlled environment chambers. Pots positions were randomly arranged. Samples of each genotype were collected from multiple flowers and plants for clearing, immunolabelling and fluorescence intensity measurements.
Blinding	Investigators were not blinded to plant genotypes during experiments. The research materials are plants so the blinding design is not easily applicable to this system. However, fluorescence tests and phenotypic observations were independently verified by colleagues using a anonymised blinding system.

Reporting for specific materials, systems and methods

We require information from authors about some types of materials, experimental systems and methods used in many studies. Here, indicate whether each material, system or method listed is relevant to your study. If you are not sure if a list item applies to your research, read the appropriate section before selecting a response.

Materials & experimental systems

n/a	Involved in the study	
☐	☒	Antibodies
☒	☐	Eukaryotic cell lines
☒	☐	Palaeontology and archaeology
☒	☐	Animals and other organisms
☒	☐	Clinical data
☒	☐	Dual use research of concern
☐	☒	Plants

Methods

n/a	Involved in the study	
☒	☐	ChIP-seq
☒	☐	Flow cytometry
☒	☐	MRI-based neuroimaging

Antibodies

Antibodies used	anti-methyl-esterified homogalacturonan LM20 (Kerafast, ELD003, 1:100 dilution);<br>anti-AGP JIM13 (Kerafast,ELD025, 1:100 dilution)<br>anti-callose BS400-2 (LAMP2H12H7, Biosupplies Australia, 1:100 dilution)<br>anti-H3K27me1 (ThermoFisher Scientific, Cat No. 49-1012, 1: 600 dilution);<br>AlexaFluor 555 anti-rat IgG (Invitrogen, Cat No. A48263, 1:200 dilution);<br>AlexaFluor 488 anti-rabbit IgG (Invitrogen, Cat No. A32731, 1:400 dilution);<br>AlexaFluor 488 anti-mouse IgG (Invitrogen, Cat No. A-11001, 1:400 dilution);<br>anti-DIG-AP (Roche, Cat No. 11093274910; 1: 5,000 dilution).
Validation	The commercial anti-pectin has been validated in several plant cell wall studies using immunolabelling. Product profile: https://www.kerafast.com/item/1585/anti-pectic-polysaccharide-homogalacturonan-lm20-antibody.<br><br>The commercial JIM13 anti-AGP has been validated in several plant cell wall studies using immunolabelling. Product profile: https://www.kerafast.com/productgroup/926/arabinogalactan-protein-agp-antibodies<br><br>The commercial anti-H3K27me1 has been validated in plants for immunolabelling. Product profile: https:// www.thermofisher.com/antibody/product/H3K27me1-Antibody-Polyclonal/49-1012<br><br>The commercial secondary antibodies (anti-rat/rabbit/mouse IgG) have been well validated in numerous studies.<br><br>The commercial anti-Digoxigenin-AP, Fab fragments, has been well validated for In situ mRNA hybridisation in many published papers, and the antibody profile can be found in the following link: https:// www.sigmaaldrich.com/catalog/product/roche/11093274910?lang=en&region=AU

Plants

Seed stocks	Arabidopsis Col-0 seed stocks were obtained from the Arabidopsis stock centre and maintained at the Waite Campus, University of Adelaide. pKNU:NLS-YFP, pAGO5:ER-YFP, pPIN1:PIN1-GFP, pWUS:WUS-GFP and pSTK:STK-GFP seed have previously been published and were generously provided by collaborating laboratories.
Novel plant genotypes	All transgenic plants were generated using Agrobacterium-mediated floral dip transformation. A minimum of 10 independent lines were maintained from each transformation experiment and at least three of these were characterised in detail. In all cases, T1, T2 and T3 lines were confirmed to display heritable phenotypes and/or expression patterns. Marker genes were crossed into the pKNU:GLUC background and ovules from the F3 genertion +/- pKNU:GLUC were compared to assess differences in expression.
Authentication	Line identity was confirmed by genotyping PCR to detect the presence of the respective transgenes and/or by localised expression of YFP, GFP or mSTR. Ovule phenotyping was also used to screen for pKNU:GLUC and pWUS:GLUS plants showing FG abortion phenotypes.
