# Supplementary material for: Germline β−1,3-glucan deposits are required for female gametogenesis in Arabidopsis thaliana
Source: Nat Commun. 2024 Jul 12;15:5875. doi: 10.1038/s41467-024-50143-0 (PMC11245613; doi:10.1038/s41467-024-50143-0)
Supplement: Supplementary file 3 — Description of Additional Supplementary Files [file 41467_2024_50143_MOESM3_ESM.pdf]

## Description of Additional Supplementary Files:

**Supplementary Dataset 1:** List of Differentially Expressed Genes (DEGs) identified in the cell-typespecific transcriptome comparisons

**Supplementary Dataset 2:** List of Gene Ontology (GO) terms enriched in the cell-type-specific transcriptome comparisons

**Supplementary Dataset 3:** List of Differentially Expressed Genes (DEGs) identified in the pKNU:GLUC and Col WT RNAseq transcriptome comparisons

**Supplementary Dataset 4:** List of Gene Ontology (GO) terms enriched in the pKNU:GLUC and Col WT transcriptome comparisons
